# Life in the desert: The impact of geographic and environmental gradients on genetic diversity and population structure of *Ivesia webberi*

**DOI:** 10.1002/ece3.8389

**Published:** 2021-11-23

**Authors:** Israel T. Borokini, Kelly B. Klingler, Mary M. Peacock

**Affiliations:** ^1^ Ecology, Evolution and Conservation Biology Graduate Program Department of Biology University of Nevada, Reno Reno Nevada USA; ^2^ University and Jepson Herbaria Department of Integrative Biology University of California, Berkeley Berkeley California USA; ^3^ Department of Environmental Conservation University of Massachusetts Amherst Massachusetts USA; ^4^ Department of Biology University of Nevada, Reno Reno Nevada USA

**Keywords:** central marginal hypothesis, gene flow, isolation by distance, isolation by environment, *Ivesia webberi*, species‐genetic diversity

## Abstract

For range‐restricted species with disjunct populations, it is critical to characterize population genetic structure, gene flow, and factors that influence functional connectivity among populations in order to design effective conservation programs. In this study, we genotyped 314 individuals from 16 extant populations of *Ivesia webberi*, a United States federally threatened Great Basin Desert using six microsatellite loci. We assessed the effects of Euclidean distance, landscape features, and ecological dissimilarity on the pairwise genetic distance of the sampled populations, while also testing for a potential relationship between *I*. *webberi* genetic diversity and diversity in the vegetative communities. The results show low levels of genetic diversity overall (*H*_e_ = 0.200–0.441; *H*_o_ = 0.192–0.605) and high genetic differentiation among populations. Genetic diversity was structured along a geographic gradient, congruent with patterns of isolation by distance. Populations near the species’ range core have relatively high genetic diversity, supporting in part a central‐marginal pattern, while also showing some evidence for a metapopulation dynamic. Peripheral populations have lower genetic diversity, significantly higher genetic distances, and higher relatedness. Genotype cluster admixture results suggest a complex dispersal pattern among populations with dispersal direction and distance varying on the landscape. Pairwise genetic distance strongly correlates with elevation, actual evapotranspiration, and summer seasonal precipitation, indicating a role for isolation by environment, which the observed phenological mismatches among the populations also support. The significant correlation between pairwise genetic distance and floristic dissimilarity in the germinated soil seed bank suggests that annual regeneration in the plant communities contribute to the maintenance of genetic diversity in *I*. *webberi*.

## Graphical abstract

One image was uploaded with the submission, which was tagged "not for review and publication." The photograph illustrates *Ivesia webberi*, the study species during the flowering season.

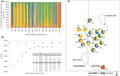

## 1 INTRODUCTION

Anthropogenic activities that lead to habitat fragmentation and loss represent some of the greatest threats to terrestrial biodiversity (Lander et al., 2019; Lughadha et al., 2020). Loss and fragmentation reduce habitat area as well as available resources, create edge effects, alter gene flow, and increase genetic differentiation among populations, which can impact plant–animal interactions especially the obligate mutualisms that facilitate pollination and seed dispersal (Aguilar et al., 2019; Fontúrbel & Murúa, 2014; Lander et al., 2019). Moreover, biogeography theory predicts that when faced with climate change, plant species can either acclimate, adapt, migrate, or go extinct (Corlett, 2016; Panetta et al., 2018). The lack of mobility in plants limits their response to environmental changes and human‐altered landscapes to either adaptation or extinction (Corlett, 2016; Panetta et al., 2018). Ultimately, the ability of plant species to adapt to environmental changes will be tied to the underlying genetic resources within populations, which, in turn, are influenced by both gene flow and population size (Barrett & Schluter, 2008; Hughes et al., 2008). A reduction in gene flow among populations can result in significant spatial genetic structure, increased selfing in self‐compatible species, genetic drift, and inbreeding. This can result in fitness costs related to inbreeding depression and reductions in fecundity, seedling survival, and ultimately population viability, as well as losses of neutral and adaptive genetic diversity (Lander et al., 2019; Nevill et al., 2019). Therefore, effective species conservation must consider how habitat protection can be designed to facilitate intraspecific population‐level functional connectivity, given that gene flow is fundamental for maintaining genetic variation and thus the evolutionary potential (Auffret et al., 2017; Spear et al., 2010). From a conservation perspective, it is critical to understand the effects of habitat fragmentation on threatened species, identify the drivers of genetic structure, and assess the capacity of populations and species to respond to future changes (Cruzan, 2001; Razgour et al., 2019; Rybicki et al., 2020). Such empirical findings can be used to facilitate functional connectivity (Neville et al., 2016) and define evolutionarily significant units (Brown et al., 2016; Peacock & Dochterman, 2012).

An isolation by distance (IBD) hypothesis predicts gene flow to be spatially patterned such that genetic differences increase with geographic distance (Jenkins et al., 2010; Wright, 1943). Similarly, the central‐marginal hypothesis (CMH) predicts reduced genetic variation and gene flow and increased pairwise genetic differentiation among populations toward the edge of the species range (Eckert et al., 2008; Micheletti & Storfer, 2015; Pfenninger et al., 2011). Indeed, spatial and latitudinal gradients in genetic diversity have been reported in many studies (Eckert et al., 2008; Pironon et al., 2016). However, other factors acting at different spatial and temporal scales can also influence the distribution of genetic variation and rates of gene flow across the landscape (Anderson et al., 2010). On fragmented landscapes, IBD alone may not fully explain the barriers to gene flow because anthropogenic activities and landscape heterogeneity can severely impact dispersal events (Gaddis et al., 2016; Spear et al., 2010) resulting in isolation by resistance (IBR; McRae, 2006; McRae & Beier, 2007). Other factors including vegetative structure, biotic interactions, elevation, rivers, mountain ranges, and anthropogenic features such as roads, urban settlements, and agricultural landscapes can also act as barriers to gene flow (Luque et al., 2012; Ortego et al., 2012). There are always exceptions, however, and some species appear to leverage human activities to enhance gene flow and expand their ranges (Auffret & Cousins, 2013; Everman & Klawinski, 2013). Furthermore, historical and current environmental conditions can exert different forms of selection pressure on populations across ecological gradients, which may result in local adaptation to divergent micro ecological conditions and increased genetic differentiation among populations. These processes can impede successful dispersal from other populations due to adaptive, phenotypic, and phenological mismatches, a condition described as isolation by environment (IBE; Sexton et al., 2014; Wang & Bradburd, 2014). Therefore, incorporating information on ecological niche and landscape heterogeneity can improve models, thereby allowing a more accurate interpretation of genetic structure and gene flow patterns and identification of barriers to functional connectivity among populations (Anderson et al., 2010; Zeller et al., 2012). However, assessing the effects of anthropogenic activities, physical features, and ecological conditions on genetic variation and functional connectivity requires a landscape genetic approach (Balkenhol et al., 2009). This is particularly important for plant species where functional connectivity is complex, given the passive nature of plant propagule dispersal (Sork & Smouse, 2006).

The Great Basin Desert is a cold desert that receives most of its annual precipitation in the winter (Comstock & Ehleringer, 1992). In addition to historical climate change, anthropogenic activities over the past 150 years have resulted in land‐cover changes, impacted wildfire regimes, and facilitated colonization by invasive and non‐native species, all of which have altered desert vegetative communities (Morris & Rowe, 2014; Wisdom et al., 2005). Moreover, temperature increases of between 0.7 and 1.4°C have already been recorded for the Great Basin Desert (1985–2011) (Snyder et al., 2019; Wagner, 2003), which may be associated with other climate changes including the decline in snowpack (Mote et al., 2005), early arrival of spring season, and dramatic interannual variation in precipitation (Baldwin et al., 2003; Chambers, 2008). In fact, depending on whether any climate mitigation strategies are enacted, these temperature increases could reach between 2 and 5 °C in the region over the next 100 years, which may increase the colonization and invasion success of the non‐native C_4_ grasses and further impact wildfire regimes in the Great Basin Desert (Smith et al., 2000; Westerling et al., 2006). For these reasons, the *Artemisia* spp. (sagebrush) ecosystem of the Great Basin Desert is one of the most critically endangered habitats in the United States (Noss et al., 1995; Stein et al., 2000), with over 600 native plants considered species of conservation concern (The Nature Conservancy, Nachlinger et al., 2001). However, the geological history, topographic complexity, and significant microclimatic gradients of the Great Basin Desert (Cassel et al., 2009; Kraft et al., 2010) offer excellent model systems for estimating the effects of natural and anthropogenic landscape features on gene flow (Davis et al., 2008), as well as the effects of historical climatic cycles on demography, the maintenance of genetic variation, and occurrence of genetic bottlenecks in native species of the Great Basin Desert. Additionally, investigating the genetic structure of desert‐dwelling plant species can elucidate factors that enhance resilience under harsh conditions. The desert ecosystem also offers a great opportunity to assess the effects of temperature and water, limiting factors in desert ecosystems, both of which can impact genetic diversity in species through increased biotic interactions in terrestrial ecosystems (Moya‐Laraño, 2010).

**FIGURE 1:**
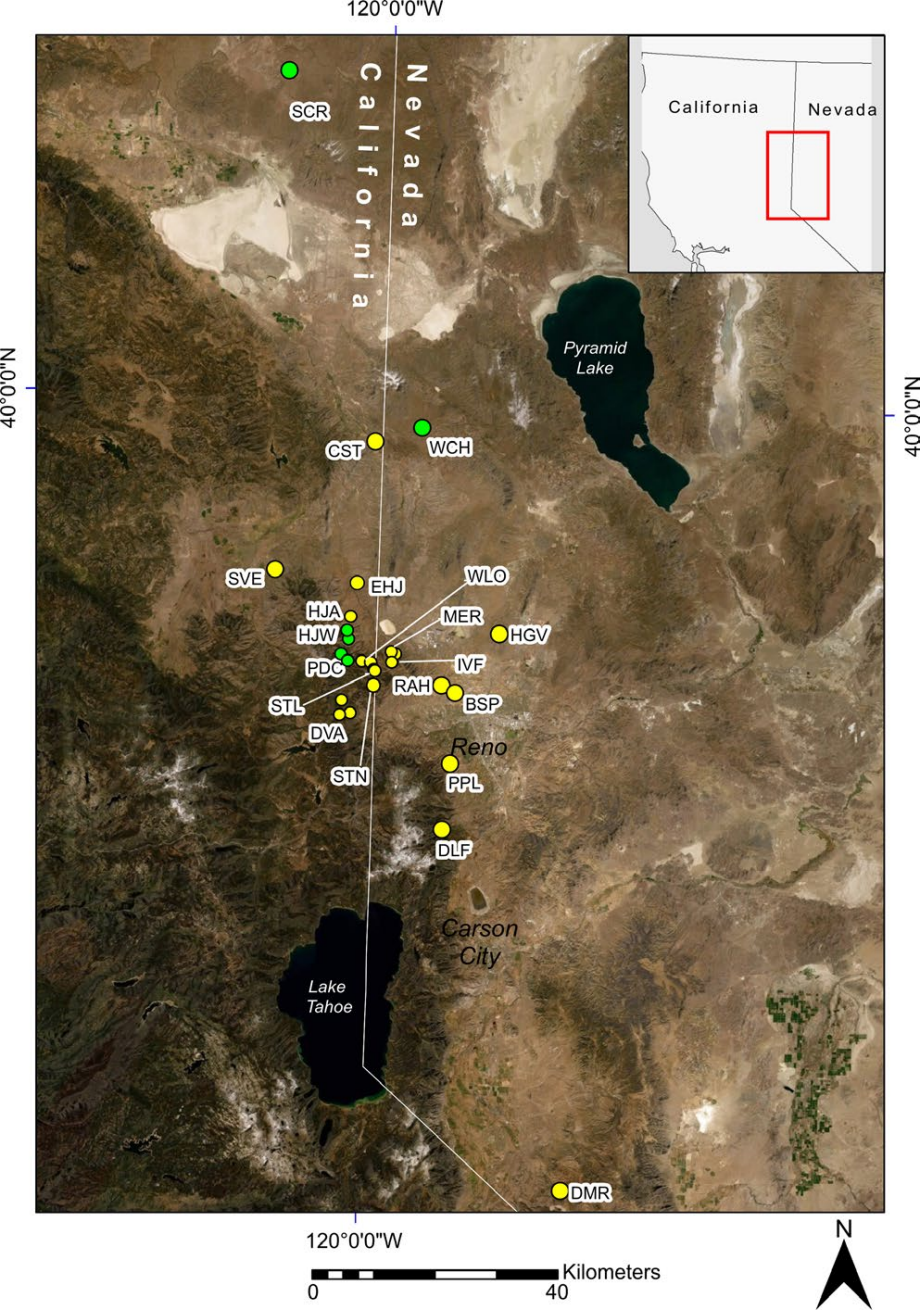
Map of the global distribution of *Ivesia webberi*. Symbols represent the geographic center of extant, mapped occurrences. Locations represented by yellow circles show the sampled populations used for this study, while green circles represent the new locations discovered after sample collections and thus not included in this study. Circle size is an artifact to avoid overlapping of locations on the map

*Ivesia webberi* A. Gray, belonging to the Rosaceae family, is a federally listed threatened (United States Endangered Species Act 1973 ESA; 16 U.S.C. § 1531 et seq.) perennial forb. Though its historical range is unknown, the species is now narrowly distributed along the western edge of the Great Basin Desert, near the Sierra Nevada Mountain Range (Figure 1; United States Fish & Wildlife Service [USFWS], 2014). Most of the populations close to the center of the species range are spatially aggregated. Vegetative regeneration from dormant root caudices and new recruitment from seed germination have been observed. The small bright yellow flowers produced by *I*. *webberi* are visited by native Hymenopterans, Dipterans, and Lepidopterans, and therefore, the species is thought to be entomophilous, but the pollinators and mating system for the species have not yet been formally identified (USFWS, 2014). The species produces dry indehiscent achene fruits that abscise into rock crevices, which are characteristic of the soil surface in all observed sites (USFWS, 2014; Witham, 2000). Indehiscent achene fruits are not adapted for long range dispersal, and we are not aware of any seed dispersal vectors for this species from field observations or peer‐reviewed literature. However, water‐assisted seed dispersal patterns via spring snowmelt and summer precipitation have been reported for other *Ivesia* species that do not reproduce vegetatively (e.g., *I*. *tweedyi*, Moseley, 1993; *I*. *lycopodioides* var. *scandularis*, Pollak, 1997). Localized seed dispersal to bare‐soil microsites, due to gravity‐assisted surface runoff from summer precipitation, likely results in seedling recruitment and colonization of decommissioned roads in many of the sites where *I*. *webberi* is found. Therefore, we expect gene flow among *I*. *webberi* populations to be more successful from pollen than from seeds (Ennos, 1994). The populations of *I*. *webberi* are located in mid‐elevation sites, which have been impacted by severe historical and current disturbance including livestock grazing, wildfires, urban settlements, off‐highway vehicle use, and climate change, where they are also threatened by habitat loss from biological invasion of alien weeds, such as *Bromus tectorum*, *Taeniatherum caput*‐*medusae*, and *Poa bulbosa* (USFWS, 2014).

We used genetic data to test hypotheses of isolation by distance, by resistance, and by environment, in addition to the species‐genetic diversity hypothesis, which posits a relationship between genetic diversity and the floristic dissimilarity (Kahilainen et al., 2014; Whitlock, 2014). We collected data on polymorphic nuclear microsatellite genetic markers: (a) to measure levels of genetic diversity, estimate effective population size (*N*_e_), and the rate and probable direction of gene flow for *I*. *webberi* populations; (b) to estimate the effect of Euclidean distance, landscape features, and ecological dissimilarity on the genetic structure in the sampled populations; and (c) investigate a relationship between pairwise *I*. *webberi* genetic diversity and floristic diversity in the vegetative communities in order to assess potential impacts of non‐native and invasive species on maintenance of genetic diversity. Due to the spatial configuration of these populations, we also (d) tested the central‐marginal hypothesis (CMH), which predicts decreased gene flow and increased pairwise genetic differentiation among populations towards the edge of the species range (Micheletti & Storfer, 2015). Despite the challenges associated with modeling plant landscape genetics due to their sedentary life, and passive seed and pollen dispersal, plant species offer an excellent opportunity to explore species’ interactions with the landscape (Alvarado‐Serrano et al., 2019; Cruzan & Hendrickson, 2020). Furthermore, plants like *I*. *webberi* with short generation times are expected to respond quicker to environmental and landscape changes; these effects can be observed in the distribution of genetic variation within the species (Aguilar et al., 2008). Moreover, identification of species‐specific threats remains critical to conservation efforts (Visconti et al., 2016), especially for range‐restricted and threatened species that are already vulnerable to genetic, environmental, and demographic stochasticity (Schwartz et al., 2006).

## 2 MATERIALS AND METHODS

### 2.1 Study species

*Ivesia webberi* is a spring blooming perennial forb which produces ~25 cm‐diameter clusters of small greenish‐gray leaves at ground level and small bright yellow flowers. Flowering occurs between May and June, while seed abscission and senescence occur simultaneously within two months of flowering. Occupied sites are sparsely vegetated flat, bench, or terrace locations in shallow, rocky, clay‐containing soils, located at elevations between 1364 and 1900 m (USFWS, 2014). Patch occupancy and size of the mapped locations of *I*. *webberi* vary between 0.05 and 71.58 acres, which suggests varying micro ecological conditions in these locations (Table 1). Preliminary flow cytometric analysis and karyotyping reveal that the species is diploid (2*n* = 2x = 28 chromosomes; Borokini, 2021). However, despite the lack of empirical information on the breeding system and pollinators of *I*. *webberi*, gene flow is thought to be more likely a result of pollen movement among populations than from seed dispersal (Ennos, 1994). However, it has not yet been established if the *I*. *webberi* floral insect visitors are pollinators. Nevertheless, foraging distance and hence potential pollen dispersal in some Hymenopterans range from 200 m to 6 km (Albrecht et al., 2009; Pasquet et al., 2008).

**TABLE 1:** *Ivesia webberi* populations sampled for this study, abbreviated (abr) site names, patch size (acres), sample size (N), mean number of alleles per locus (*N*_a_), allelic richness over all loci per population (*R*_T_), and mean observed (*H*_o_) and expected (*H*_e_) heterozygosity per population

Population	Abr	Patch size	N	Averages			
				*N*_a_	*R*_T_	*H*_o_	*H*_e_
Sierra Valley	SVE	44.8	21	2.67	13.45	0.400	0.344
Constantia	CST	1.91	20	2.17	10.31	0.192	0.204
Evans Canyon, East of Hallelujah junction wildlife area (HJWA)	EHJ	0.14	24	2.17	11.60	0.419	0.305
HJWA	HJA	0.05	18	2.67	14.56	0.410	0.363
Dog Valley meadow	DVA	71.58	22	2.67	12.83	0.359	0.317
White Lake overlook	WLO	13.56	22	3.33	15.63	0.487	0.405
Mules Ear Flat	MER	0.14	20	3.00	15.28	0.559	0.441
Ivesia flat	IVF	0.73	20	2.83	14.29	0.605	0.435
Stateline road 1	STL	7.03	9	2.50	14.29	0.495	0.379
Stateline road 2	STN	4.03	13	2.33	12.85	0.316	0.346
Hungry valley	HGV	0.16	24	2.50	12.28	0.492	0.369
Black springs	BSP	6.31	18	2.33	11.85	0.315	0.271
Raleigh heights	RAH	9.55	23	3.17	13.88	0.423	0.355
Dutch Louie flat	DLF	1.35	19	2.83	12.51	0.237	0.242
The Pines power line	PPL	0.14	18	2.17	10.79	0.265	0.216
Dante Mine Road	DMR	0.56	23	1.83	9.34	0.274	0.200

### 2.2 Sample collection, DNA extraction, PCR amplification, and genotyping

Five leaves were collected per plant from 24 randomly selected plants in each of the 16 sampled *I*. *webberi* populations (Table 1). The leaves were stored in paper collection bags with silica gel to facilitate drying of samples at room temperature. GPS coordinates of each sample were also recorded using Garmin eTrex 20×.

Five mg of leaf tissue from each plant sample (*n* = 384) were processed using a TissueLyser II (QIAGEN Inc., Valencia, CA, USA). Genomic DNA was extracted using the protocol described in the DNeasy96 Plant Extraction Mini kit (QIAGEN). DNA per sample was quantified at the Nevada Genomics Center (https://www.unr.edu/genomics) using the PicoGreen dsDNA assay (Thermo Fisher, Waltham, MA, USA). DNA concentration was determined using a standard curve equation following DNA detection under the Fluoroskan Microplate Fluorometer (Thermo Fisher, Waltham, MA, USA).

No microsatellite loci have been developed for *I*. *webberi* nor for any of species in this genus. We initially tested 20 microsatellite loci developed from *Potentilla pusilla* (Dobeš & Scheffknecht, 2012) for use with *I*. *webberi*. *Potentilla* is phylogenetically related to *Ivesia* (Töpel et al., 2012) and the developed markers were reported to be polymorphic and cross‐amplified with other species at success rates ranging from 86% to 97% (Dobeš & Scheffknecht, 2012). Of these 20 loci, six polymorphic microsatellite loci amplified consistently in *I*. *webberi* and were further optimized for this study (Appendix S1). PCR amplification was carried out in a Labnet International Inc. MultiGene^TM^ OptiMax thermal cycler (115V model) in 10.0 µl reaction volumes in a 96‐well format using the Qiagen Multiplex PCR kit, which contains HotStarTaq DNA polymerase, dNTPs, and PCR buffer at a 2× concentration. Loci were amplified in single or multiplexed PCRs with a final concentration of 0.05 μM of each tailed forward primer and 0.1 μM of each reverse primer. Each PCR included 5 µl of Multiplex Mix, 20 ng of DNA, between 0.1–0.2 µl of primer and approximately 4.8 µl of ultrapure molecular grade water. PCR parameters included a 15‐minute hot start at 95°C, then 41 cycles of 95°C for 30 s, followed by a touchdown annealing temperature that ranged between 65 to 55°C for 90 s with a final elongation step of 72°C for 30 s. The touchdown annealing temperature begins with 7 cycles at 65°C, 7 cycles at 61°C, 7 cycles at 58°C, and 20 cycles at 55°C.

PCR products were diluted to an appropriate concentration and 1 µl of diluted PCR product was added to 19 µl of Hi‐Di Formamide/LIZ500 size standard (Applied Biosystems, ABI). Fragment analysis was done on an Applied Biosystems (ABI) Prism 3730 DNA analyzer at the Nevada Genomics Center (https://naes.unr.edu/genomics). All alleles generated were scored, binned, and genotyped using the ABI GeneMapper software (version 5; Applied Biosystems, Thermo Fisher Scientific). We also re‐amplified 30% of the sample (~115 samples) to validate genotyping reliability. Individual leaf samples that failed to amplify were removed from the analysis, thus reducing the sample size from 384 to 314 (Table 1).

### 2.3 Genetic analyses

#### 2.3.1 Population‐level diversity metrics

We used FSTAT 2.9.4 (Goudet, 1995) to test for Hardy–Weinberg equilibrium (HWE) across all loci, calculate the number of alleles (*N*_a_), allelic richness (*R*_S_), the inbreeding coefficient (*F*_IS_), and to determine whether linkage disequilibrium among loci was present within populations. The outcrossing rate (*t*) was calculated using the inbreeding coefficient (*F*_IS_) and the formula *t* = (1–*F*_IS_)/(1 + *F*_IS_) (Weir, 1996). We estimated genetic diversity (*H*_e_, *H*_o_) using Microsatellite Toolkit in Excel. MICROCHECKER v.2.2.3 (van Oosterhout et al., 2004) was used to test for allelic dropout and null alleles. Preferential amplification of shorter alleles (Wattier et al., 1998) can result in what appears as a deficit of heterozygotes, which is used to indicate large allelic dropout. To check for this, MICROCHECKER employs several null allele estimators, including the Chakraborty et al. (1992) estimator for null alleles, two Brookfield (1996) estimators, and the van Oosterhout (2004) estimator. We used HP‐Rare (Kalinowski, 2005) to quantify private alleles per locus per population. Relatedness (*r*) among individuals within populations was calculated using the Lynch and Ritland (1999) equations in GenAlEx v.6.5 (Peakall & Smouse, 2012). We tested for genetic bottlenecks per population using BOTTLENECK v.1.2.02 (Piry et al., 1999) and the single step (SMM) and two‐phase (TPM) mutation models.

#### 2.3.2 Population genetic structure

We used GenAlEx to estimate pairwise genetic differentiation among populations (*F*_ST_) and calculate the number of migrants between populations (*N*_m_) based upon *F*_ST_ estimates. STRUCTURE (v.2.3.4; Pritchard et al., 2007) was run to estimate the number of Bayesian genotype clusters (*K*) across all *I*. *webberi* populations, using a 100,000‐iteration burn‐in followed by ten 500,000 Markov chain Monte Carlo (MCMC) replications per *K*, for *K *= 1–10. The optimal number of genotype clusters was determined using the Δ*K* method (Evanno et al., 2005). We conducted AMOVA in GenAlEx to characterize the partitioning of genetic variation on the landscape. Principal coordinate analysis (PCoA) was conducted using *F*_ST_ values to investigate population structuring (Jombart et al., 2009; Sant’Anna et al., 2020); using the *pcoa* function in the ape R package (Paradis & Schliep, 2019; R Development Core Team, 2020). Effective population size (*N*_e_) was calculated for each population and Bayesian genotype cluster identified using the linkage disequilibrium (LD) method in NeEstimator v.2.0 (Do et al., 2014). *N*_e_ for the genotype clusters identified using STRUCTURE was calculated using individuals with a *Q* > 0.8, where *Q* is the probability of assignment to an individual genotype cluster (Pritchard et al., 2000, 2007).

#### 2.3.3 Isolation by distance and landscape resistance

We assessed the effects of geographical distance (isolation by distance; IBD), land‐cover, inverse of habitat suitability (isolation by resistance; IBR), and ecological dissimilarity (isolation by environment; IBE) on pairwise genetic distance among the 16 *I*. *webberi* populations. Both IBD and IBR models were fitted using a linear mixed effects model framework in the ResistanceGA R package v. 4.1‐11 (Peterman, 2018). Additionally, IBD was also investigated using the Mantel test. Slatkin's linearized pairwise *F*_ST_ values, which account for microsatellite mutation following the single step model (Di Rienzo et al., 1994; Slatkin, 1995), were used as the response variable. Pairwise geographical distance was estimated using the great–circle distance method that accounted for the earth's curvature, from the GPS coordinates of the polygon centroid for each population (Rosenmai, 2014). Land cover was derived from the Multi‐Resolution Land Characteristics (MRLC) development of the U.S. National Land‐cover Database (NLCD) 2016 (Xian et al., 2013), and the habitat suitability map was produced from ensemble projection of niche modeling replicates from six algorithms with TSS ≥ 0.7 (Appendix S2).

ResistanceGA uses a genetic algorithm from the GA R package to optimize the conversion of predictor variables into resistance surfaces and testing the effect of the parameterized resistances on gene flow (Peterman, 2018; Scrucca, 2013, 2017). The algorithm converts predictor GIS layers into resistance surfaces, calculates the pairwise effective distance (e.g., least cost path and random walk), fits maximum likelihood population effects (MLPE) models on pairwise genetic distance using the pairwise effective distance as predictor, and, finally, selects the best model to describe isolation by resistance on pairwise genetic distances (Peterman et al., 2019). The habitat suitability map was resampled to 250 m and converted to a resistance surface using an inverse monomolecular method, which assumes a negative relationship between gene flow and landscape resistance (Peterman, 2018). The land cover was also resampled to 250 m and reduced to 15 feature classes each of which was automatically assigned a resistance value, following optimization. We are aware of the potential effect of spatial resolutions on landscape connectivity modeling results, but this resampling is inevitable due to the computational limitations in running ResistanceGA (Cushman & Landguth, 2010; O’Connell et al., 2019). A composite resistance surface layer which combined both the optimized land‐cover layer and inverse habitat suitability map was also used.

Functional connectivity in the landscape was calculated using *commuteDistance* function, which is similar to the resistance estimates calculated using CIRCUITSCAPE (McRae et al., 2008). For optimal computing efficiency with parallel processing, ResistanceGA was interfaced with CIRCUITSCAPE v.5.7.1 (Anantharaman et al., 2020). Random‐walk commute‐distance estimates are preferred over the least cost path, which assumes that gene flow is maximized in the lowest cost path because individuals have knowledge of all possible paths, an assumption that is unlikely to be true (Adriaensen et al., 2013). We used default parameterizations and 10 iterations in ResistanceGA for the independent optimization of the two resistance surfaces (i.e., habitat suitability map and land‐cover layer).

The MLPE model used the linearized pairwise *F*_ST_ as the response variable, the 16 population codes as the random effect term, while the fixed effect terms included pairwise geographical distance among the populations, land‐cover resistance, and the transformed habitat suitability map. The MLPE model fitted a null model (*I*. *webberi* population ID), an IBD model (using pairwise geographical distance and population ID), and an IBR model (comprising population ID, using pairwise geographical distance and the resistance surfaces both individually and in combination). Following the 10 MLPE model replicates, we conducted bootstrapping to assess the sensitivity of the MLPE models to the spatial distribution of *I*. *webberi* populations. Here, we randomly resampled 75% of the data without replacement, fitted the MLPE models again using 10,000 iterations, and selected the best models using the average AICc values (a modification of the Akaike information criterion [AIC] for small sample sizes) and predictor weight (relative contribution of each predictor to the model). The percentage contribution of each surface within the multisurface optimization was calculated by dividing each transformed resistance surface by the sum of the composite resistance surface (Peterman, 2018).

#### 2.3.4 Isolation by environment

To investigate the effect of ecological dissimilarity on pairwise genetic distances among the 16 *I*. *webberi* population, we assembled 72 predictors representing bioclimatic, biotic, and topographic conditions in the species habitats. These predictors were reduced to seven uncorrelated (*r* > 0.6) variables following three consecutive feature reduction analyses (Appendix S2). These include cumulative actual evapotranspiration, summer seasonal precipitation, perennial herbaceous vegetative cover, minimum monthly temperature, cosine aspect, Topographic Position Index, and elevation (Appendices S3 and S4). Distance matrices were generated for each of the seven predictor variables, using the Euclidean distance method, to investigate isolation by environment in *I*. *webberi*.

Mantel tests explored direct association of pairwise genetic distance and the environmental dissimilarity matrices; however, the significant spatial genetic structure necessitates accounting for geographical distance in the relationship (Kozak & Wiens, 2006). Therefore, we fitted generalized dissimilarity models (GDM; Ferrier et al., 2007) to investigate patterns of isolation by environment in the genetic structure. GDM, as implemented in the gdm R package (Fitzpatrick et al., 2021) uses I‐spline basis functions to assess the variance in the genetic distance by each of the predictor variables and uses permutation to assess the relative importance of each predictor variable, as they correspond to the maximum height of each spline (Ferrier et al., 2007; Xu et al., 2017). The full model contains all predictor variables and geographical distance, while other modeling iterations were fitted after randomly reordering the table of environmental predictors using 1,000 permutations (Ferrier et al., 2007). Model significance was assessed by comparing the deviance explained by the GDM iteration to the deviance explained by the full and unpermuted GDM (Ferrier et al., 2007). A similar permutation was used to assess the significance of variable importance to the model. Here, all predictor variables are permuted one at a time, using a backward elimination method, in GDM iterations, while variable weight (importance) is determined as the percent change in the deviance explained in the GDMs with and without the variable (Ferrier et al., 2007).

#### 2.3.5 Central‐marginal hypothesis

The range center of the *I*. *webberi* was estimated using the range center index (RCI; Enquist et al., 1995) method based on the latitudinal decimal degrees of the population sites. In the RCI sites closer to the species’ range center have values closer to zero, the northernmost site was assigned the value of 1, while the southernmost site was assigned a value of −1. Pearson correlation test between *I*. *webberi* RCI and allelic richness and mean observed heterozygosity (*H*_o_), both of which are indicators of genetic diversity, was used to investigate the predictions of the central‐marginal hypothesis. Additionally, Mantel test was used to investigate the relationship between a matrix of the latitudinal degrees and the pairwise genetic distance (*F*_ST_) among the sampled populations.

#### 2.3.6 Relationship between plant community diversity and *Ivesia webberi* genetic diversity

We tested the species‐genetic diversity hypothesis, which posits that a relationship exists between *I*. *webberi* genetic diversity and the floristic dissimilarity across the sampled sites (Kahilainen et al., 2014; Whitlock, 2014). In a separate study (Borokini et al., 2021), species richness, abundance, and diversity of both the aboveground plant communities and the soil seed bank of 10 of the 16 sites were quantified (Appendix S5). Here, we conducted separate Spearman correlation tests between genetic diversity (i.e., allelic richness and mean observed heterozygosity), and floristic richness and diversity of both the aboveground vegetation and the soil seed bank in each of the 10 sites. Species diversity was the exponential conversion of the Shannon–Weiner H’ index for each site (i.e., the effective number of species; Jost, 2006). Additionally, we assessed a relationship between linearized pairwise *F*_ST_ and the floristic dissimilarity matrix in both the aboveground vegetation and the soil seed bank for the 10 sampled sites, using separate Mantel tests, each with 10,000 permutations. Throughout the study, Mantel tests were conducted in ECODIST R package (Goslee & Urban, 2007). To account for the effect of geographic distance, we fitted separate multiple regressions on distance matrices (MRM; Lichstein, 2007) between pairwise *F*_ST_ genetic distance and floristic dissimilarity matrices (β‐diversity) of the aboveground flora and the soil seed bank across the 10 sites. The floristic dissimilarity matrices were generated using the Bray–Curtis method. MRM analysis was conducted with 10,000 permutations in the phytools R package (Revell, 2012).

## 3 RESULTS

### 3.1 Population‐level genetic diversity metrics

We genotyped 314 *I*. *webberi* individuals at six polymorphic nuclear microsatellite loci (Appendix S6). Allelic diversity per locus (*N*_a_) ranged from 3–13 alleles, while allelic richness (correction for sample size) per locus (*R*_S_) ranged from 2.002 to 4.073 (Appendix S6). In addition, we found private alleles at multiple loci for each sampling site (Appendix S6). No locus showed evidence of null alleles or allelic dropout. Two loci were out of HWE in single or multiple populations. Locus PMS1694 had a significant positive *F*_IS_ in CST, which is the northernmost population sampled (*F*_IS_ = 0.898, *p *= .0005), indicating a heterozygote deficit. Locus PMS1438 had significant negative *F*_IS_ values in multiple populations indicating heterozygous excess (SVE, EHJ, DVA, WLO, MER, IVF, HGV, BSP, RAH, PPL, DMR; *F*_IS_ range = −0.8 to −1.0; *p *= .0005) (Appendix S6). Five of the populations with significant negative *F*_IS_ values at the PMS1438 locus were also peripheral populations (SVE, DVA, HGV, PPL, and DMR; Figure 1). Genetic bottlenecks were observed for both the TPM and SMM mutation models in five populations, four of which had significant negative *F*_IS_ values (**bolded**) (**EHJ** – TPM *p *= .017, SMM *p *= .017; **MER** – TPM *p *= .042, SMM *p *= .047; **BSP** – TPM *p *= .037, SMM *p *= .039; **DMR** – TPM *p *= .02, SMM *p *= .02; STL – TPM, *p *= .016, SMM, *p *= .023; Table 2). We reran the bottleneck analysis after removing PMS1438 from the dataset to test whether the significant *F*_IS_ values at this locus were driving the significant bottleneck results. We found evidence for genetic bottlenecks under the SMM model at BSP (*p *= .039), WLO (*p *= .011) and RAH (*p *= .015), while the MER population was close to being significant (*p *= .088), suggesting a potential bottleneck (Table 2). These results suggest that negative *F*_IS_ values at PMS1438 contributed to the bottleneck results, but do not appear to solely account for observed patterns.

**TABLE 2:** *p* Values per population for genetic bottlenecks under the two phase (TPM) and single step (SMM) mutation models for 6 and 5 loci (PMS1438 removed). Bolded values are significant

Population	Population code	TPM	SMM	SMM 5 loci
Sierra Valley	SVE	0.145	0.174	0.092
Constantia	CST	0.163	0.147	0.208
Evans Canyon, East of Hallelujah junction wildlife area (HJWA)	EHJ	**0.017**	**0.018**	0.646
HJWA	HJA	0.135	0.146	0.186
Dog Valley meadow	DVA	0.140	0.162	0.084
White Lake overlook	WLO	0.139	0.132	**0.011**
Mules Ear Flat	MER	**0.042**	**0.047**	0.088
Ivesia flat	IVF	0.114	0.126	0.301
Stateline road 1	STL	**0.016**	**0.022**	0.424
Stateline road 2	STN	0.118	0.130	0.576
Hungry valley	HGV	0.104	0.117	0.522
Black springs	BSP	**0.037**	**0.040**	**0.039**
Raleigh heights	RAH	0.146	0.153	**0.015**
Dutch Louie flat	DLF	0.137	0.151	0.141
The Pines power line	PPL	0.110	0.124	0.096
Dante Mine Road	DMR	**0.020**	**0.020**	0.231

Allelic richness summed over all loci (*R*_T_) per population was the highest in WLO (*R*_T_ = 15.63), which is located in the cluster of populations at the center of *I*. *webberi* range (Table 1; Figure 1). The lowest value was found in the isolated southernmost population sampled (DMR, *R*_T_ = 9.34; Table 1). Similarly, the isolated northernmost population sampled (CST) also had lower allelic diversity (*R*_T_ = 10.31; Table 1). Low levels of mean expected and observed heterozygosities (*H*_e_ = 0.200–0.441; *H*_o_ = 0.192–0.605) per population were observed. Similar to the distribution of allelic richness per population, expected and observed heterozygosities were the highest among centrally located populations, but the lowest in the peripheral populations (Table 1). We conducted pairwise t tests between *H*_e_ and *H*_o_ for all loci across populations. The only significant difference between *H*_e_ and *H*_o_ was for the PMS1439 locus, which had significantly higher observed heterozygosity than expected (*p *< .0001).

**FIGURE 2:**
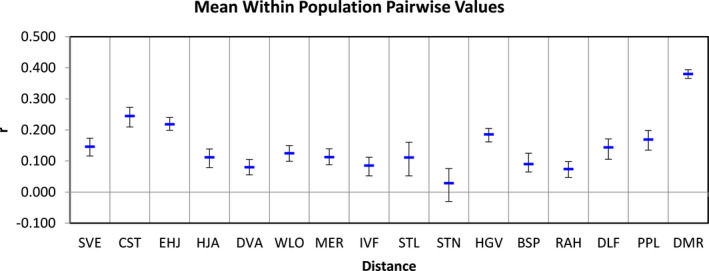
Lynch and Ritland (1999) mean relatedness (*r*) ±SD for each the 16 sampled *Ivesia webberi* populations. Mean relatedness represents mean within population pairwise values

In addition, DMR and CST, peripheral populations, had the highest levels of within‐population relatedness (*r* = 0.38 and *r* = 0.25, respectively; Figure 2), while most of the centrally located and spatially proximate populations had low levels of *r* (Figures 1 and 2). Because the confidence intervals for most of the population *N*_e_ estimates (69%) included infinity, we do not report those values here. For the populations that we could calculate both an *N*_e_ and 95% CI, the values ranged from 0.9 to 11.6 (Table 3). We also calculated *N*e for each of the five genotype clusters identified below (Table 3).

**TABLE 3:** Effective population size for the populations where we could calculate a 95% CI and for the genotype clusters (including only individuals with *Q* ≥ 0.8 per cluster). *N*_e_ values reported here were calculated using the linkage disequilibrium method

	*N*_e_	95% CI
Populations		
HJA	0.9	0.6–1.3
DVA	5.2	3.3–8.5
STN	3.3	1.6–7.7
DLF	8.3	4.4–20.0
RAH	11.7	6.8–24.1
Genotype clusters		
1 (orange)	2.6	1.7–4.0
2 (gray)	27.9	12.6–148.1
3 (yellow)	40.5	19.5–162.1
4 (blue)	18.3	10.5–35.9
5 (green)	20.4	12.3–37.6

### 3.2 Population genetic structure

Pairwise *F*_ST_ values among the sampled *I*. *webberi* populations tended to be high and statistically significant (Table 4; corrected *p *= .0004). The nonsignificant pairwise *F*_ST_ values were found primarily among the spatially proximate populations at the center of the range. The global *F*_ST_ was 0.158 (equivalent to *θ*, Weir & Cockerham, 1984). The most isolated population sampled (DMR) was significantly differentiated from all remaining populations. Pairwise *N*_m_ values suggest low rates of gene flow among populations on average, with the majority of *N*_m_ values ranging between 1 and 4 (Table 4). For the southernmost population DMR, all *N*_m_ values were <1. There were a number of population pairs that had *N*_m_ values >5 (range 5–19). The majority of these population pairs were separated by <5 km. However, the distance between population pairs with *N*_m_ ranging from 8 to 19 ranged from 7 to18 km. The population pairs with high *N*_m_ values were primarily among core populations and populations on the eastern edge of the distribution. These data suggest that there may be extant intervening populations that have not been sampled or have been recently extirpated that could represent stepping stones for dispersal among more distantly spaced populations. Dispersal among spatially distant populations may also be facilitated by specific environmental conditions or unidentified dispersal agents. Analysis of molecular variation (AMOVA) showed that 71% of the molecular variance was within individuals, while 11% and 18% of the molecular variance were among individuals and populations respectively (Table 5), and the outcrossing rate was estimated to be 87.3%.

**TABLE 4:** Pairwise genetic differentiation (*F*_ST_) values among the 16 *Ivesia webberi* populations (below the diagonal) and gene flow (*N*_m_) values (above the diagonal). Raw pairwise *F*_ST_ values were reported here, while linearized *F*_ST_ values were used for all analyses; bold values indicate statistical difference (corrected *p *= .0004). *N*_m_ values ≥6 are also bolded

Population	SVE	CST	EHJ	HJA	DVA	WLO	MER	IVF	STL	STN	HGV	BSP	RAH	DLF	PPL	DMR
SVE	–	0.481	0.431	0.651	0.917	0.735	1.137	1.021	0.919	1.459	0.890	0.760	1.082	2.112	2.138	0.529
CST	**0.657**	–	2.458	2.127	1.647	3.064	1.159	2.698	1.704	1.891	1.238	1.399	1.764	0.982	0.865	0.386
EHJ	**0.135**	**0.770**	–	4.748	2.104	1.948	0.945	1.512	3.081	1.872	1.212	1.410	1.278	0.892	0.769	0.299
HJA	**0.114**	**0.612**	0.045	–	2.634	3.666	1.320	1.894	3.954	3.178	1.476	1.705	1.431	1.464	1.193	0.376
DVA	**0.156**	**0.354**	**0.121**	**0.104**	–	2.128	1.991	2.538	**16.688**	4.540	2.023	**19.148**	3.968	**5.559**	3.833	0.473
WLO	0.041	**0.541**	**0.109**	0.084	**0.106**	–	2.476	3.053	4.088	3.852	1.368	1.503	1.541	1.483	1.295	0.453
MER	**0.227**	**0.337**	**0.285**	**0.248**	**0.090**	**0.156**	–	**5.605**	2.937	3.545	1.640	1.307	1.963	3.434	4.097	0.489
IVF	**0.130**	**0.317**	**0.220**	**0.160**	**0.102**	0.090	0.048	–	3.069	4.297	**8.522**	2.403	**12.505**	3.085	2.810	0.558
STL	**0.195**	0.440	**0.109**	**0.105**	0.020	0.087	0.107	0.137	–	na	1.851	2.796	2.533	4.314	2.465	0.514
STN	**0.143**	**0.287**	**0.159**	0.126	0.049	0.108	0.120	0.092	0.021	–	2.439	2.657	3.177	**6.724**	3.254	0.961
HGV	**0.252**	**0.335**	**0.257**	**0.188**	**0.146**	**0.188**	**0.131**	**0.028**	**0.186**	**0.124**	–	2.463	**11.515**	2.194	1.831	0.518
BSP	**0.167**	0.352	**0.164**	**0.121**	0.008	**0.110**	0.099	**0.070**	**0.072**	0.040	**0.102**	–	**6.363**	3.692	2.861	0.404
RAH	**0.184**	**0.224**	**0.228**	**0.170**	0.059	**0.141**	0.073	0.015	0.116	0.053	0.025	0.027	–	3.840	3.594	0.591
DLF	**0.352**	0.201	**0.407**	**0.282**	0.068	**0.262**	0.107	**0.132**	**0.154**	0.102	**0.167**	**0.053**	0.066	–	0.000	0.296
PPL	**0.421**	0.216	**0.465**	**0.352**	0.101	**0.316**	0.104	**0.148**	0.220	0.153	**0.185**	0.080	0.077	0.000	–	0.507
DMR	**0.770**	**0.667**	**0.992**	**0.861**	**0.631**	**0.709**	**0.606**	**0.531**	**0.609**	**0.319**	**0.561**	**0.687**	**0.477**	**0.612**	**0.738**	–

**TABLE 5:** Analysis of molecular variance (AMOVA) of the genetic variation among and within 16 *Ivesia webberi* populations

Source of variation	df	SS	MS	Estimated variance	Percent contribution
Among populations	15	151.280	10.085	0.225	16
Among individuals	298	381.928	1.282	0.082	6
Within individuals	314	351.000	1.118	1.118	78
Total	627	884.209		1.425	100

**FIGURE 3:**
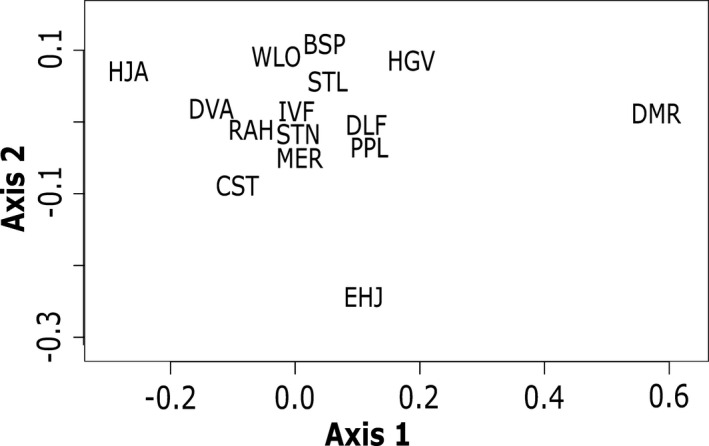
A plot of the principal coordinate analysis (PCoA) of the pairwise *F*_ST_ genetic distance for the 16 sampled *Ivesia webberi* populations

The PCoA based on the pairwise genetic differentiation (*F*_ST_) validates the genetic distance in the DMR population from the rest of the sampled populations (Figure 3). Furthermore, a high genetic similarity, suggesting gene flow, between DLF and PPL, and among IVF, MER, and STN populations were confirmed (Figure 3). Overall, the majority of the populations near the center of *I*. *webberi* distribution range were clustered together, while the peripheral populations, especially the DMR, were isolated in ordination space (Figure 3). A barplot of the PCoA eigenvalues show that axis 1, distantly followed by axis 2, accounted for most of the variance in the pairwise *F*_ST_ (Appendix S7).

**FIGURE 4:**
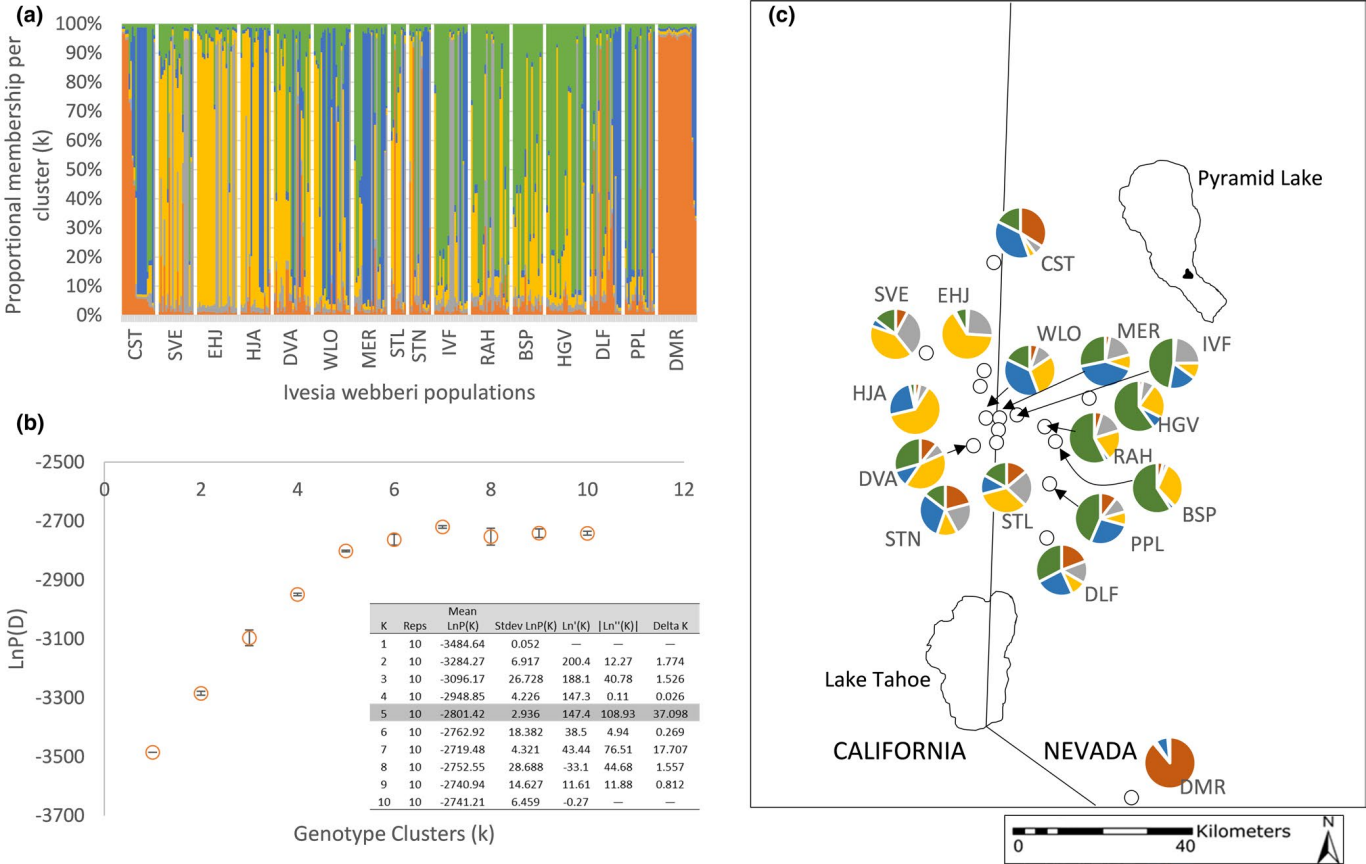
(a) STRUCTURE output showing proportional membership per genotype cluster (*K* = 5) per individual. Populations were arranged by north‐to‐south latitude. (b) The natural log of the probability of the data [LnP(D)] values per *K* for *K* = 1–10 (inset Δ*K* for *K* = 1–10). (c) Pie graphs of genotype cluster membership per sampling location for individuals with *Q* ≥ 80%

Five genotype clusters (*K*) were identified as the best fit of the data [Average LnP(D) = −2801.42, SD± = 2.936, Δ*K* = 37.098] (Figure 4). Individuals in the isolated DMR population at the southern end of the distribution assigned to a single genotype cluster (orange) (Figure 4). Interestingly, some of the individuals in the northernmost CST population as well as a few additional individuals from other populations also had high proportional membership in this genotype cluster. Populations with individuals assigned to the orange cluster tended to be oriented northwest to southeast with few if any individuals from the easternmost populations having assignment to this cluster. Individuals assigned to the blue and gray genotype clusters also tended to be in populations found at the center of the range; however, CST and DMR populations also had individuals assigned to these clusters. The two genotype clusters with the greatest proportional membership and spatial extent were the yellow and green genotype clusters. The westernmost populations had the highest assignment to the yellow genotype cluster, and there was little admixture among genotype clusters observed within individuals in these populations (Figure 4). Assignment in the yellow genotype cluster gradually declined moving eastward with increasing assignment to the green genotype cluster. We did, however, observe more admixture between the green and yellow genotype clusters in the eastern populations that had high proportional assignment in the green genotype cluster suggesting contemporary gene flow (Figure 4). No individuals from the CST or DMR populations assigned to the yellow or green genotype clusters. Effective population size was the largest for genotype cluster 3 (yellow; *N*_e_ = 40.5) and lowest in genotype cluster 1 (orange; *N*_e_ = 2.6; Table 3).

### 3.3 Drivers of genetic structure

Pairwise linearized *F*_ST_ shows a significant geographical pattern (Mantel *r* = 0.860, *p* < .001) among the 16 sampled *I*. *webberi* populations indicating isolation by distance and significant spatial genetic structure (Table 6). Similar results were produced in the MLPE model showing that geographic distance explained most of the variance in genetic distance among the 16 *I*. *webberi* populations, based on the model weight and AICc parameters (Table 7).

**TABLE 6:** Results of the Mantel tests, multiple regression on distance matrices (MRM) analysis, and generalized dissimilarity models (GDM) between pairwise genetic distance (*F*_ST_) and geographical distance, environmental variables, and floristic dissimilarity among the sampled *Ivesia webberi* populations. MRM results show the model fit (*R* ^2^), regression coefficients (β), and *p* values for each of the floristic dissimilarity matrices, while GDM results show the regression coefficients (β), relative importance (weight), and *p* values for each predictor variable. Mantel tests were run in ECODIST R package, MRM analysis was conducted in phytools R package, both implemented with 10,000 permutations, while GDMs were fitted in the gdm R package with 1,000 permutations for the variable importance analysis

Predictors	Mantel test		MRM			GDM		
	*r*	*p*	*R* ^2^	β	*p*	β	Weight	*p*
Geographical distance	0.860	<.001	0.738	0.523	<.001	0.623	3.087	<.001
Aboveground species dissimilarity^a^	−0.047	.542	0.696	0.470	<.001	n/a	n/a	n/a
Soil seed bank species dissimilarity^a^	0.960	<.001	0.879	0.405	<.001	n/a	n/a	n/a
Actual evapotranspiration	0.633	.006	n/a	n/a	n/a	0.082	3.331	.044
Cosine aspect	0.182	.147	n/a	n/a	n/a	0.000	0.000	.956
Summer seasonal precipitation	0.726	.004	n/a	n/a	n/a	0.280	1.738	.112
Minimum monthly temperature	−0.063	.604	n/a	n/a	n/a	0.038	0.328	.425
Perennial herbaceous cover	−0.086	.617	n/a	n/a	n/a	0.026	0.434	.379
Topographic Position Index	−0.147	.817	n/a	n/a	n/a	0.030	0.498	.332
Elevation	0.266	.105	n/a	n/a	n/a	0.210	5.809	.048

^a^ Species dissimilarity in both the aboveground vegetation and the soil seed bank were computed from 10 of the 16 *I*. *webberi* populations (Borokini et al., 2021). Therefore, pairwise genetic distance (*F*_ST_) corresponding to the sampled 10 populations was used.

**TABLE 7:** Summary table from the bootstrap analysis on the MLPE models with 10,000 iterations in ResistanceGA R package. *k* is the number of parameters fitted in the bootstrap analysis, AIC and AICc represent average values of the two parameters in the bootstrap analysis, LL is the average log likelihood of the bootstrap analysis. Weight represents the average contribution of each predictor to the model relative to all predictors included. *R* ^2^m is the average marginal *R* ^2^ value of the bootstrap analysis on the MLPE model

Parameters	Land cover:niche	Land cover	Niche	Distance	Null
*K*	19	16	4	2	1
AIC	−64.1803	−70.3673	−91.0559	−96.2079	n/a
AICc	695.8197	473.6327	−85.3417	−94.8746	n/a
LL	51.0902	51.1836	49.5279	50.1039	n/a
*R* ^2^m	0.55184	0.5616	0.4934	0.4855	n/a
Weight	0.0000	0.0000	0.0488	0.9512	n/a

Genetic diversity was generally higher in the centrally located populations than in the peripheral populations. However, despite this spatial genetic diversity pattern, we did not observe a significant relationship between range center index (RCI) and allelic richness (Spearman's correlation ρ = .393, *p* = .132) or observed heterozygosity (Spearman's correlation ρ = .257, *p* = .337). In contrast, we found a significant positive relationship between pairwise latitudinal degrees and genetic distance among the 16 populations (Mantel *r* = 0.849, *p *< .001).

The results of the maximum likelihood population effects (MLPE) models and the bootstrap analysis showed that land cover, representing urban settlements and highways, did not pose any barrier to gene flow among *I*. *webberi* populations (Table 7). The majority of the variance in the genetic distance was explained by geographical distance (AICc = −94.875, weight = 0.951), followed by the inverse of habitat suitability projection map (AICc = −85.342, weight = 0.049; Table 7). Therefore, these results do not support an isolation by resistance, but rather validate an isolation by distance pattern given the genetic differentiation among *I*. *webberi* populations.

**FIGURE 5:**
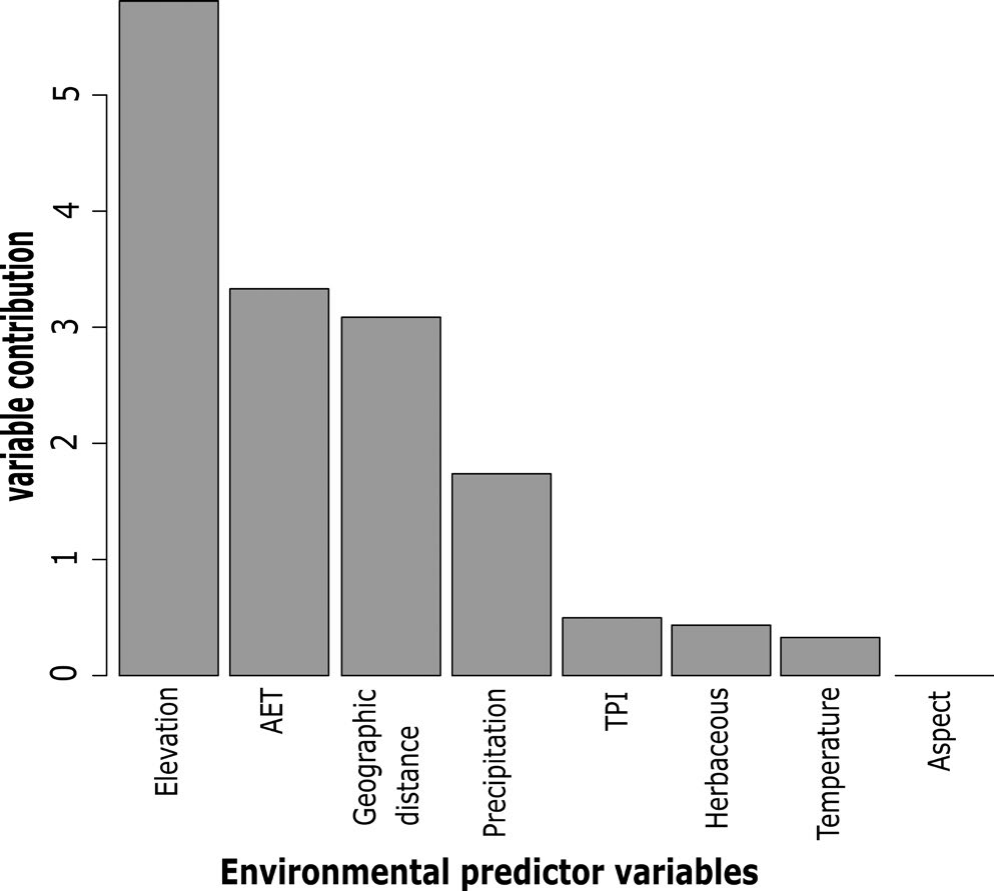
A plot of the relative importance of the seven environmental predictor variables and geographic distance on *Ivesia webberi* genetic structure from the generalized dissimilarity model. AET stands for the cumulative actual evapotranspiration, precipitation represents summer seasonal precipitation, TPI stands for Topographic Position Index, herbaceous means Perennial herbaceous vegetative cover, temperature stands for minimum monthly temperature, and aspect represents cosine aspect

In addition to the isolation by distance pattern, the results provide support for an isolation by environment. The results of the Mantel test and the GDMs explain the relationship between genetic distance and dissimilarity matrices of ecological predictor variables (Table 6). Mantel tests show a significant relationship only between *I*. *webberi* genetic distance and the dissimilarity matrices for actual evapotranspiration (AET) and summer seasonal precipitation (Table 6), whereas GDMs showed significant relationships between *F*_ST_ and geographical distance, AET, and elevation, respectively (Table 6). The variable importance analysis also revealed that these three variables contributed the most to the patterns of genetic structure in *I*. *webberi* (Figure 5). All GDMs had a significant fit to the data (*p *< .001) and accounted for more than 50% of the deviance in the data structure, with three GDMs explaining 76% of the deviance (Table 6).

### 3.4 Relationship between floristic diversity and genetic diversity in *Ivesia webberi*

Species richness and diversity in the aboveground vegetative communities as well as the soil seed bank diversity showed a positive trending relationship with genetic diversity (allelic richness and observed heterozygosity) of *I*. *webberi*, in contrast to soil seed bank richness which has a negative relationship with both genetic diversity parameters (Table 8). However, these relationships were not statistically significant (*p *< .05; Table 8). There was no relationship between genetic distance and aboveground floristic dissimilarity from the Mantel test results, but MRM results did show a significant relationship (Table 6). The soil seed bank species dissimilarity among the 10 sites showed a significant relationship with the pairwise genetic distance for both Mantel test and the MRM analysis (Table 6).

**TABLE 8:** Spearman's correlation *rho* between metrics of alpha diversity in 10 vegetative communities harboring *Ivesia webberi* and their corresponding genetic diversity. Allelic richness represents mean allelic richness, and *H*o stands for observed heterozygosity in each of the 10 sampled populations

Community alpha diversity	Allelic richness	*p*	*H*_o_	*p*
AGV species richness	0.585	.075	0.354	.316
AGV species diversity	0.394	.263	0.055	.892
SSB species richness	−0.120	.742	−0.044	.904
SSB species diversity	0.139	.707	0.418	.232

## 4 DISCUSSION

The evolutionary potential of species under changing environmental pressures is strongly tied to the maintenance of genetic variation, which can be directly tied to gene flow and connectivity among populations. The results of this study reveal contrasting patterns of significant population genetic structure and isolation in addition to dispersal and gene flow among the sampled *I*. *webberi* populations. We found evidence of isolation by distance, by environment and by resistance as well as environmental correlates of standing genetic variation. These patterns appear to be largely driven by geographic distance, where complementary analyses (Mantel test, GDM, and MLPE models) provide strong support for the isolation by distance model, but some of the variance is also explained by evapotranspiration and precipitation, and to a smaller degree by latitudinal gradient and habitat suitability.

Population levels of mean observed heterozygosity tended to be low (0.390) ranging from 0.192 to 0.605, with the exception of two neighboring populations at the center of the range (MER and IVF), which had higher observed heterozygosity (0.559 and 0.605, respectively). Not surprisingly, the highest levels of heterozygosity and allelic richness as well as nonsignificant pairwise *F*_ST_ estimates were found among spatially proximate populations at the center of the range. Evidence of both genetic bottlenecks and high levels of genetic variation among centrally located populations suggest that these populations may have a metapopulation dynamic defined by an extinction‐colonization patch dynamic (Hanski, 1999) and genetic coalescence (Gilpin, 1991), as well as a stepping stone dispersal dynamic among extant patches (Peacock & Smith, 1997).

However, the Bayesian genotype clustering analysis reveals a more complex movement pattern. Membership in the individual genotype clusters was not confined to specific populations, but was spread among multiple populations across the species range supporting movement among the spatially discrete sites. We see a gradual decrease in assignment to the yellow genotype cluster in the western portion of the range and increased membership in the green cluster moving from west to east consistent with a pattern of isolation by distance. However, the easternmost populations (BSP, HGV, RAH) have few or no individuals that assign to the other genotype clusters (orange, blue, and gray). Individuals which assign to blue genotype cluster are found primarily in the three most centrally located populations (Figure 4; WLO, MER, STN), but membership in this genotype cluster appears to trend north to south with assignment found among individuals in centrally located populations, but also in both the northernmost and southernmost populations (CST and DMR). The orange genotype cluster also appears to have a north‐to‐south distribution with the highest membership found in the northernmost and southernmost populations (CST and DMR). The differing spatial patterns observed for the genotype clusters suggests multiple influences on patterns of dispersal including both pollen and seed dispersal, which may be in play with landscape features influencing which dispersal mode is most prevalent among populations.

Gene flow via pollen transfer may occur by native Dipterans, Lepidopterans, and/or Hymenopterans, which have been observed to be visiting *Ivesia* flowers frequently during field surveys (Auffret et al., 2017; Dick et al., 2008). The isolation by distance patterns may therefore be partially explained by the flight ranges and foraging behavior exhibited by these potential pollen vectors (Matter et al., 2013; Mokany et al., 2014). However, it is unknown at this point whether the floral visitors on *I*. *webberi* are effective pollinators. Although we did observe admixture between the yellow and green genotype clusters as cluster membership changed from yellow to green moving west to the east, suggestive of pollen movement. Gamete dispersal (pollen) would result in pollination and hence admixture, whereas seed dispersal would not. Only through future sexual reproduction would dispersed seeds colonizing a new population lead to admixture. Once seeds are established and if the adult plant reproduces vegetatively, no admixture would be observed and distinct genotype cluster assignments within populations would persist. Individuals which assign to the blue, gray, and orange genotype clusters show very little evidence of admixture. *I*. *webberi* is known to reproduce vegetatively, which could explain the high proportional membership of individuals in the same population to distinct genotype clusters. In fact, negative *F*_IS_ values for some of the loci, indicating a heterozygous excess, in multiple locations, together with high within individual genetic variation is consistent with vegetative regeneration and clonality in *I*. *webberi* (Balloux et al., 2005). The levels of genetic diversity observed in this study are also similar to those observed in mixed‐mating plants and outcrossing species (e.gCulley & Wolfe, 2001; Meeus et al., 2012), which suggests there is both successful sexual reproduction as well as vegetative reproduction occurring in *I*. *webberi* populations (Dlugosch & Parker, 2008; Genton et al., 2005; Muller et al., 2011). Mixed mating systems have been reported in over 42% of flowering plants (Goodwillie et al., 2005) and previous studies show that most of the genetic variance is within populations for such species, while self‐compatible species maintain a large proportion of their genetic diversity among populations (Nybom, 2004). Furthermore, outcrossing species generally have low‐to‐moderate genetic differentiation; hence, they can exhibit dramatic genetic responses to geographic isolation (Hamrick & Godt, 1996). This is consistent with what we have observed in *I*. *webberi*, where adjacent populations have moderate‐to‐high gene flow, while isolated populations have higher genetic differentiation and low dispersal rates. However, other life‐history traits such as pollen and seed dispersal, population density, life span, and geographic distribution can have a great impact on population genetic diversity in species (Edwards et al., 2021; Huang et al., 2019). For example, short‐lived and prolific species have relatively high genetic diversity (Leimu et al., 2006; Nybom, 2004). Past and current climatic conditions and other ecological factors also have dramatic effects on the spatial genetic structure of species (Alvarez et al., 2009). For example, glacial refugia and postglacial dispersal have shaped spatial genetic structure in many species (Hewitt, 2000; Petit et al., 2002).

The spatial genetic structure of *I*. *webberi* appears to be driven by the genetic isolation observed for the peripheral populations and evidence that is at least suggestive of a metapopulation type dynamic among the centrally located populations. As a result, we did not find support for the predictions of the central‐marginal hypothesis (Spearman rank correlation revealed positive but nonsignificant associations between genetic diversity estimates and the range center index), but rather we found evidence of a complex interplay among isolation by distance, by environment, and by resistance. Isolation by resistance was driven by the inverse of the projected habitat suitability, not land cover. This indicates that potentially suitable areas from the niche models may play an important role in genetic structure and among population gene flow as undiscovered populations may act, or did act if currently extirpated, as stepping stones for gene flow among more spatially distant populations as suggested by estimates of *N*_m_. This study showed that land cover may be a less important driver of genetic structure in this species, which may be partly due to the fact that the habitat suitability map has already explained the isolation by resistance pattern that occurs within the land‐cover layer. Moreover, most urban settlements within *I*. *webberi's* range are in lower elevations, whereas *I*. *webberi* populations are found in higher elevations and forest vegetation, which are under federal and state protections. Theoretically, pollinator‐driven gene flow among *I*. *webberi* populations would not pass through the unsuitable urban landscape. This is particularly true for the spatially aggregated populations in the center of the species’ range.

Genetic differentiation also has a significant positive relationship with pairwise difference in actual evapotranspiration across all analyses. Elevation and precipitation were shown to have significant relationships with genetic distance (Mantel test and GDM respectively). This highlights significant ecological dissimilarity among the sites which correlates with genetic distance and may indicate isolation by environment. Both actual evapotranspiration and precipitation represent water availability and climatic stress, challenges to persistence for native flora in the Great Basin Desert. The differences in water availability among these sites may be attributed to their varying elevation and topographic positions which also determine the duration of their exposure to sunlight. Field observations suggest that *I*. *webberi* have responded to these varying microclimatic conditions across the sites through variation in phenology. For example, populations at lower elevations were observed to regenerate earlier than those in the higher elevations and this could result in a temporal mismatch in flowering which can impede successful gene flow via pollen transfer among the populations. Previous studies also show significant influence of water availability, temperature, and precipitation on genetic diversity in different plant species (Oliveira et al., 2018; Smith et al., 2020; Tso & Allan, 2019). Moreover, climatic resistance to gene flow has been reported for plant species, and this may be attributed to climatic effect on the physiology of probable pollen vectors of *I*. *webberi* (Alvarado‐Serrano et al., 2019). Previous studies focusing on connectivity among populations of animal species report strong movement costs of climatic resistance surfaces, which were attributed to their physiological tolerance limits (Flores‐Manzanero et al., 2019; Hohnen et al., 2016; Sexton et al., 2014).

Most of the analyses between species richness and diversity with genetic diversity and effective population size estimates, for the plant communities at the subset of 10 *Ivesia webberi* sites, revealed positive but not significant (*p* > .05) results, which may be attributed to the small sample size (*n* = 10). Similarly, species beta‐diversity of the soil seed bank, but not aboveground flora, was significantly associated with the pairwise genetic distance. Most of the sites where *I*. *webberi* is located have undergone varying degrees of anthropogenic habitat modifications, including one site that served as part of a trail for westward expansion of European colonizers during the late 19th and early 20th centuries. Moreover, these sites have been affected by frequent wildfires and non‐native and invasive plants. However, field observation and natural history indicate that this species has high potential for recovery postdisturbance, which may be linked to the dormant deep taproot caudices buried and firmly protected in the argilic subsurface soil horizon. This may explain field observations that suggest that the abundance of the invasive plant species does not prevent the annual vegetative regeneration of established matured *I*. *webberi* individuals. However, invasive alien species can hinder new recruitment of native plants by outcompeting the young and delicate seedlings (Borokini et al., 2021; Chambers et al., 2007). Therefore, a significant relationship between beta diversity in the soil seed bank and the population genetic distance in the 10 surveyed sites may reflect effects of differing microhabitat conditions that affect seed‐based recruitment of *I*. *webberi* into the population. Furthermore, this significant relationship underscores the role of the soil seed bank in maintaining the genetic diversity of native species (Mandák et al., 2012; Schulz et al., 2018). This finding is congruent with previous studies that show a significant and positive relationship between genetic diversity and floristic community structure (Hughes et al., 2008; Kahilainen et al., 2014; Vellend et al., 2014). Interspecific competition in niche space within an ecological community, therefore, could impact both neutral and adaptive genetic diversity in populations over time and trigger varying selection across different populations within the species (Bailey et al., 2009; Vellend, 2005; Whitlock, 2014). Intraspecific genetic diversity, in turn, can influence community responses to environmental changes and determine the velocity of shifts in community structure and functions (Broadhurst et al., 2008; Whitlock, 2014).

The results of this study show relatively high genetic diversity for the populations near the center of *Ivesia webberi* distribution range, with moderate gene flow and relatively low differentiation among adjacent populations. In contrast, the peripheral populations are geographically and genetically isolated and may already be experiencing genetic drift and inbreeding. Therefore, conservation strategies should include efforts to facilitate functional connectivity of the DMR and CST populations with the rest of the populations. This study also increased the scientific understanding of *Ivesia webberi* natural history by establishing that the species is a mixed mating and facultative out crosser, with greater likelihood for pollen‐based gene flow patterned both by geographical distance and by environment. This finding is congruent with existing literature and meta‐analysis of 70 studies that showed that gene flow among plants was more commonly patterned along a combination of isolation by distance and by environment, respectively (Sexton et al., 2014). In the light of these findings, conservation efforts must also consider the effects of gradual encroachment of residential developments into higher elevations on potential insect‐assisted pollen transfer among *I*. *webberi* populations because insects avoid human‐altered landscapes (Delnevo et al., 2020; Làzaro et al., 2020). Anthropogenic landscape features result in potential habitat loss and fragmentation, which could increase extirpation risks and resistance to gene flow among the populations. Furthermore, the significant isolation by environment pattern in the genetic structure of *I*. *webberi* validates concerns that regional climate change, characterized by milder winters, hotter summers, and increased variability between low and higher elevations in the Great Basin Desert (Mote et al., 2005), may further exacerbate phenological mismatches and hence greater population genetic differentiation along an elevation gradient. Furthermore, conservation efforts on *I*. *webberi* should strive to include genetic characterization of newly discovered sites and investigation of dispersal dynamics as well as protection and monitoring of potential movement corridors in addition to active control of invasive alien species.

## CONFLICT OF INTEREST

The authors declare no competing interests.

## AUTHOR CONTRIBUTION

**Israel T. Borokini:** Conceptualization (equal); Data curation (lead); Formal analysis (lead); Funding acquisition (equal); Investigation (equal); Methodology (lead); Software (lead); Validation (equal); Visualization (equal); Writing‐original draft (lead); Writing‐review & editing (supporting). **Kelly B. Klingler:** Data curation (equal); Formal analysis (equal); Methodology (equal); Software (equal); Writing‐review & editing (equal). **Mary M. Peacock:** Conceptualization (equal); Funding acquisition (equal); Project administration (lead); Supervision (equal); Visualization (equal); Writing‐original draft (equal); Writing‐review & editing (lead).

## Supporting information

Appendix S1‐S7

## Data Availability

Data generated or analyzed during this study are included in this article and the supplementary information files. The microsatellite loci used in this study are already published in the Dobeš and Scheffknecht (2012) paper. The raw genotype scores for the 314 sampled *Ivesia webberi* individuals are accessible at Knowledge Network for Biocomplexity: https://doi.org/10.5063/F1KK997V.
