# Supplementary material for: Life in the desert: The impact of geographic and environmental gradients on genetic diversity and population structure of Ivesia webberi
Source: Ecol Evol. 2021 Nov 23;11(23):17537–56. doi: 10.1002/ece3.8389 (PMC8668734; doi:10.1002/ece3.8389)
Supplement: Supplementary file 1 — Appendix S1‐S7 [file ECE3-11-17537-s001.docx]

**APPENDICES**

**Appendix S1.** Locus, primer sequences, repeat motif and PCR melting temperatures for the six microsatellite loci used in this study.

| Locus | Primer 5’-3’ | Repeat  Motif | Melting  temperature (°C) |
| --- | --- | --- | --- |
| PMS 1080 | F: AAATAGGCCATCCCAATTCC  R: TGCCCATCTTTCTTCTGGTT | (TAG)_14_ | 66.7  57.9 |
| PMS 1180 | F: CGATCGTAACCGTTCTCCAT  R: ACCGCTCTTCTTCTCCGATT | (TC)_4_, (GGC)_7_ | 65.3  58.5 |
| PMS 1438 | F: GGACTTGGGACTTTGTTGGA  R: TCCCAAATGCAATCGTGTAA | (AC)_10_ | 68.5  56.6 |
| PMS 1665 | F: CCAAGTGAAGGAAGCCAAAC  R: GCCGACGAAGAAGGAAGAC | (AG)_6_ | 68.2  59.7 |
| PMS 1694 | F: CCTCGAGGAACAACCTGTTT  R: CATGGACTGAGGAAGAACACAA | (AT)_13_ | 67.3  58.8 |
| PMS 2190 | F: ATAAAGGCAACGCAAGATCA  R: CGTATAATCTTACCAATCAATTAAACA | (CA)_5_/(TA)_16_ | 65.5  54.8 |

*The loci were developed from *Potentilla pusilla* and cross amplified with *I. webberi* (Dobeš & Scheffknecht, 2012). See Appendix S6 for the number of genotyped individuals, alleles observed, allelic richness, expected and observed heterozygosity, and inbreeding coefficient per locus across the 16 *I. webberi* populations

**References**

Dobeš, C., & Scheffknecht, S. (2012). Isolation and characterization of microsatellite loci for the *Potentilla* core group (Rosaceae) using 454 sequencing. *Molecular Ecology Resources,* **12**(4), 726–739. https://doi.org/10.1111/j.1755-0998.2012.03134.x

**Appendix S2.** Fitting of the ecological niche modeling for *Ivesia webberi*.

A total of 72 predictor variables describing edaphic, topographic, land-cover, vegetative cover, climatic and biotic factors were assembled for fitting niche models for *Ivesia webberi*. However, in order to avoid multicollinearity, the predictor variables were reduced to six uncorrelated predictors using a combination of Kendall *r* correlation coefficient, feature selection runs in Boruta R package (Kursa & Rudnicki, 2010), and recursive feature elimination algorithm in caret R package (Kuhn, 2008). The selected predictor variables were described in Appendices S3 and S4.

Niche model was fitted for *I. webberi* using 31 occurrence points and 102 spatially thinned true absence points, using six algorithms, including Generalized linear models (GLM), Generalized additive models (GAM), Boosted Regression Trees (BRT), Random Forests (RF), Maximum Entropy (MAXENT), and Artificial Neural Networks (ANN). The spatial dataset was a product of iterative niche modeling and successive field validation to the projected suitable sites from 2015 to 2020. The models were fitted in BIOMOD2 R package (Thuiller et al., 2009), using the default settings. GLM were generated using quadratic terms in stepwise approach, and using the Akaike information Criterion (AIC) for model selection. BRT models were generated by fitting 2,500 trees and three cross-validations, while GAM was analyzed with a spline smoothing function. We used five cross-validations to select the optimal hidden layers in ANN models, we fitted 500 random forest models, and ran MAXENT models with 10,000 background points, using linear, quadratic and product features but with logistic model output. Due to the small size of occurrence points, spatial data was not partitioned to independent training and test data, rather all niche models were fitted with 10 replicates each for the six algorithms using 80% of the data for cross-validation (Araújo et al., 2005; Thuiller et al., 2009).

Model performance was measured using True statistic skill (TSS; Allouche et al., 2006), area under the curve (AUC) of the receiver operated characteristics (ROC) plot (Hanley & McNeil, 1982), specificity, and Continuous Boyce index (CBI; Boyce et al., 2002) to produce model predictions with maximal accuracy and minimal omission errors. Model replicates with (TSS≥0.7) were used for ensemble modeling from which projection of habitat suitability maps were produced.

**References**

Allouche, O., Tsoar, A., & Kadmon, R. (2006). Assessing the accuracy of species distribution models: Prevalence, kappa and the true skill statistic (TSS). *Journal of Applied Ecology*, **43**(6), 1223–1232. https://doi.org/10.1111/j.1365-2664.2006.01214.x

Araújo, M. B., Whittaker, R. J., Ladle, R. J., & Erhard, M. (2005). Reducing uncertainty in projections of extinction risk from climate change. *Global Ecology and Biogeography*, **14**(6), 529–538. https://doi.org/10.1111/j.1466-822X.2005.00182.x

Boyce, M. S., Vernier, P. R., Nielsen, S. E., & Schmiegelow, F. K. A. (2002). Evaluating resource selection functions. *Ecological Modelling*, **157**(2-3), 281-300. https://doi.org/10.1016/S0304-3800(02)00200-4

Hanley, J. A., & McNeil, B. J. (1982). The meaning and use of the area under a receiver operating characteristic (ROC) curve. *Radiology*, **143**(1), 29–36. https://doi.org/10.1148/radiology.143.1.7063747

Kuhn, M. (2008). Building Predictive models in R using the caret package. *Journal of Statistical Software,* **28**(5), 1-26. 10.18637/jss.v028.i05

Kursa, M. B., & Rudnicki, W. R. (2010). Feature selection with the Boruta package. *Journal of Statistical Software,* **36**(11), 1-13. 10.18637/jss.v036.i11

Thuiller, W., Lafourcade, B., Engler, R., & Araújo, M. B. (2009). BIOMOD – a platform for ensemble forecasting of species distributions. *Ecography*, **32**(3), 1-5. https://doi.org/10.1111/j.1600-0587.2008.05742.x

**Appendix S3.** Ecological conditions in the 16 *Ivesia webberi* populations based on the selected ecological variables

| Population code | AET (mm) | Cosine aspect | Summer precipitation (mm) | Temperature (°C) | Herbaceous cover | Topographic position index | Elevation (m) |
| --- | --- | --- | --- | --- | --- | --- | --- |
| SVE | 243.57 | -0.52 | 25.15 | -7.40 | 32.16 | -45.26 | 1528 |
| CST | 248.11 | 0.64 | 22.42 | -6.13 | 24.71 | -111.29 | 1363 |
| EHJ | 184.19 | 0.95 | 27.27 | -7.16 | 27.74 | -131.05 | 1537 |
| HJA | 189.89 | 0.99 | 29.32 | -7.42 | 35.11 | -102.13 | 1597 |
| DVA | 274.64 | 0.12 | 28.03 | -7.36 | 17.13 | -179.72 | 1762 |
| WLO | 218.31 | 0.35 | 28.29 | -6.76 | 15.70 | -65.21 | 1724 |
| MER | 240.00 | 0.89 | 25.99 | -6.69 | 18.06 | -114.72 | 1661 |
| IVF | 244.10 | 0.51 | 26.05 | -6.78 | 30.40 | 0.08 | 1770 |
| STL | 235.90 | 0.74 | 26.61 | -6.63 | 24.01 | -99.67 | 1716 |
| STN | 248.80 | -0.52 | 27.35 | -7.11 | 22.77 | -80.85 | 1807 |
| HGV | 215.68 | 0.86 | 22.65 | -5.04 | 23.71 | 44.45 | 1594 |
| BSP | 229.91 | 0.72 | 25.65 | -6.31 | 32.09 | 25.93 | 1708 |
| RAH | 223.13 | 0.96 | 24.39 | -5.90 | 23.46 | 25.12 | 1626 |
| DLF | 284.36 | -0.81 | 27.51 | -6.34 | 41.44 | -184.36 | 1943 |
| PPL | 241.22 | 0.93 | 26.01 | -5.88 | 50.19 | -42.74 | 1586 |
| DMR | 321.21 | -0.64 | 34.72 | -6.75 | 16.96 | -103.10 | 1805 |

AET stands for cumulative actual evapotranspiration, temperature stands for minimum monthly temperature, while herbaceous cover describes perennial herbaceous cover layer.

**Appendix S4**. A description of the selected predictor variables used to test the isolation of environment pattern in the gene flow among the 16 sampled *Ivesia webberi* populations

| Predictor name | Description and source |
| --- | --- |
| Cumulative actual evapotranspiration | Water balance variable calculated from 800 m 1971-2000 PRISM climate normals (Daly et al., 2008), available water capacity (Chaney et al., 2016), and the 1-arcsecond digital elevation model (DEM; USGS, 2017) combined in the Climatic Water Deficit Toolbox for ArcGIS (Dilts, 2014; Dilts et al., 2015). AET represents the simultaneous availability of water and energy to support plant productivity. |
| Minimum monthly temperature | The bioclimatic variables were downscaled from the PRISM climatic data (1970-2001) normals (Daly et al., 2008) from 4 km to 30 m spatial resolution, using BIOCLIM methods (Booth et al., 2014) and the climatic water deficit toolbox (Dilts, 2014; Dilts et al., 2015) |
| Summer seasonal precipitation |  |
| Cosine aspect | Cosine aspect was calculated in ArcMap version 10.6.1 using USGS (2017) DEM and the slope layers, the formula: *θ* × cos(α), where = *θ* is slope (in percentage), and α is aspect (in radians) |
| Topographic Position Index | Calculated from USGS (2017) DEM in ArcMap version 10.6.1, using formula introduced by Weiss (2001), and a 333 m neighborhood. At 333 m scale, the landscape is classified into either a valley or a mountain range |
| Perennial herbaceous cover | It is a vegetation type raster layer sourced from the Multi-Resolution Land Characteristics (MRLC) development of the U.S. National Land-cover Database (NLCD) 2016 Shrub component products (Xian et al., 2013) |
| Elevation | A 30 m (1 arc second) digital elevation model (DEM) from National elevation dataset (USGS, 2017) |

**References**

Booth, T. H., Nix, H. A., Busby, J. R., & Hutchinson, M. F. (2014). BIOCLIM: the first species distribution modelling package, its early applications and relevance to most current MaxEnt studies. *Diversity and Distributions,* **20**(1), 1-9. https://doi.org/10.1111/ddi.12144

Chaney, N. W., Wood, E. F., McBratney, A. B., Hempel, J. W., Nauman, T. W., Brungard, C. W., & Odgers, N. P. (2016). POLARIS: A 30-meter probabilistic soil series map of the contiguous United States. *Geoderma*, **274**, 54–67. https://doi.org/10.1029/2018WR022797

Daly, C., Halbleib, M., Smith, J. I., Gibson, W. P., Doggett, M. K., Taylor, G. H., Curtis, J., & Pasteris, P. A. (2008). Physiographically-sensitive mapping of temperature and precipitation across the conterminous United States. *International Journal of Climatology,* **28**(15), 2031-2064. https://doi.org/10.1002/joc.1688

Dilts, T. E., Weisberg, P. J., Dencker, C. M., & Chambers, J. C. (2015). Functionally relevant climate variables for arid lands: a climatic water deficit approach for modelling desert shrub distributions. *Journal of Biogeography*, **42**(10), 1986-1997. https://doi.org/10.1111/jbi.12561

Dilts, T.E. (2014). Climatic Water Deficit Toolbox for ArcGIS 10.1. University of Nevada Reno. Available at: http://www.arcgis.com/home/item.html?id=de9ca57d43c041148b815da7ce4aa3a0

U.S. Geological Survey [USGS] (2017). 1 Arc-second Digital Elevation Models (DEMs) - USGS National Map 3DEP Downloadable Data Collection. U.S. Geological Survey. Accessed at https://www.usgs.gov/core-science-systems/ngp/tnm-delivery/

Weiss, A. (2001). Topographic position and landform analysis. Poster presentation, ESRI User Conference, San Diego, California, USA.

Xian, G., Homer, C., Meyer, D., & Granneman, B. (2013). An approach for characterizing the distribution of shrubland ecosystem components as continuous fields as part of NLCD. *ISPRS Journal of Photogrammetry and Remote Sensing*, **86**, 136-149. https://doi.org/10.1016/j.isprsjprs.2013.09.009

**Appendix S5**. Floristic richness and diversity in the aboveground and the soil seed bank of the 10 *Ivesia webberi* sampled populations. Vegetative community data was taken from Borokini et al. (2021).

| Population | Code | Aboveground community | | Soil seed bank | |
| --- | --- | --- | --- | --- | --- |
|  |  | Species richness | Species diversity | Species richness | Species diversity |
| Constantia | CST | 18 | 3.41 | 18 | 1.38 |
| Evans Canyon, East of Hallelujah junction wildlife area (HJWA) | EHJ | 20 | 3.53 | 14 | 1.80 |
| Dog Valley meadow | DVA | 34 | 12.35 | 14 | 8.72 |
| White Lake overlook | WLO | 26 | 10.32 | 16 | 1.72 |
| Mules Ear Flat | MER | 26 | 4.07 | 16 | 2.33 |
| Ivesia flat | IVF | 22 | 6.28 | 16 | 3.05 |
| Hungry valley | HGV | 24 | 1.89 | 21 | 3.04 |
| Black springs | BSP | 25 | 3.62 | 20 | 1.33 |
| Raleigh heights | RAH | 20 | 6.26 | 18 | 1.79 |
| Dante Mine Road | DMR | 21 | 8.81 | 16 | 2.65 |

Species diversity of the exponent conversion of Shannon-Weiner H’ index into natural numbers (effective number of species; Jost, 2006)

**References**

Borokini, I. T., Weisberg, P. J., & Peacock, M. M. (2021). Quantifying the relationship between soil seed bank and plant community assemblage in sites harboring the threatened *Ivesia webberi* in the western Great Basin Desert. *Applied Vegetation Science*, **24**(1), e12547 https://doi.org/10.1111/avsc.12547

Jost, L. (2006). Entropy and diversity. *Oikos,* **113**, 363–375. https://doi.org/10.1111/j.2006.0030-1299.14714.x

**Appendix S6**. Number of individuals genotyped (*N*), number of alleles observed (A), allelic richness (*R*_S_), private allelic richness (*Pa*), expected (*H*e) and observed (*H*o) heterozygosities, and inbreeding coefficient (*F*_IS_) for each locus across the 16 *Ivesia webberi* populations. *F*_IS_ values in bold text are statistically significant (adjusted *P*=0.0005, based on 1920 randomizations). NA means no analysis. Mean allelic richness per locus per population for PMS 1080, PMS 1180, PMS 1438, PMS 1665, PMS 1694, and PMS 2190 were 2.002, 4.073, 2.088, 2.047, 3.435, and 2.192, respectively.

| Locus |  | SVE | CST | EHJ | HJA | DVA | WLO | MER | IVF | STL | STN | HGV | BSP | RAH | DLF | PPL | DMR |
| --- | --- | --- | --- | --- | --- | --- | --- | --- | --- | --- | --- | --- | --- | --- | --- | --- | --- |
| PMS 1080 | *N* | 20 | 12 | 24 | 13 | 20 | 13 | 11 | 16 | 8 | 9 | 22 | 18 | 23 | 14 | 13 | 20 |
|  | A | 2 | 1 | 2 | 2 | 2 | 3 | 3 | 2 | 2 | 2 | 2 | 2 | 2 | 1 | 1 | 2 |
|  | *R*_S_ | 1.35 | 1.00 | 2.00 | 1.92 | 1.97 | 2.34 | 2.64 | 1.44 | 2.00 | 2.00 | 1.54 | 1.63 | 1.52 | 1.00 | 1.00 | 1.74 |
|  | *Pa* | 0.07 | 0.00 | 0.00 | 0.00 | 0.00 | 0.49 | 0.27 | 0.00 | 0.00 | 0.00 | 0.13 | 0.00 | 0.00 | 0.00 | 0.00 | 0.39 |
|  | *H*e | 0.05 | 0.00 | 0.45 | 0.21 | 0.30 | 0.22 | 0.48 | 0.06 | 0.5 | 0.29 | 0.09 | 0.11 | 0.09 | 0.00 | 0.00 | 0.14 |
|  | *H*o | 0.05 | 0.00 | 0.67 | 0.23 | 0.15 | 0.23 | 0.64 | 0.06 | 0.75 | 0.33 | 0.09 | 0.11 | 0.09 | 0.00 | 0.00 | 0.15 |
|  | *F*_IS_ | 0 | NA | -0.48 | -0.09 | 0.5 | -0.06 | -0.35 | 0.00 | -0.56 | -0.14 | -0.02 | -0.03 | -0.02 | NA | NA | -0.06 |
| PMS 1180 | *N* | 21 | 19 | 24 | 18 | 22 | 22 | 19 | 20 | 9 | 12 | 24 | 18 | 23 | 17 | 17 | 22 |
|  | A | 4 | 3 | 3 | 4 | 4 | 4 | 4 | 5 | 4 | 4 | 5 | 4 | 4 | 6 | 3 | 2 |
|  | *R*_S_ | 2.60 | 1.98 | 2.47 | 3.56 | 3.28 | 3.23 | 3.14 | 3.90 | 4.00 | 3.52 | 3.45 | 3.27 | 2.82 | 3.71 | 2.31 | 2.00 |
|  | *Pa* | 0.00 | 0.14 | 0.04 | 0.88 | 0.00 | 0.01 | 0.16 | 0.33 | 0.01 | 0.00 | 0.31 | 0.22 | 0.00 | 0.49 | 0.16 | 0.01 |
|  | *H*e | 0.33 | 0.20 | 0.38 | 0.60 | 0.66 | 0.59 | 0.44 | 0.64 | 0.79 | 0.66 | 0.59 | 0.60 | 0.50 | 0.41 | 0.27 | 0.46 |
|  | *H*o | 0.29 | 0.20 | 0.38 | 0.50 | 0.59 | 0.55 | 0.21 | 0.80 | 0.89 | 0.33 | 0.54 | 0.50 | 0.52 | 0.47 | 0.24 | 0.41 |
|  | *F*_IS_ | 0.14 | 0.00 | 0.01 | 0.17 | 0.11 | 0.08 | 0.53 | -0.26 | -0.13 | 0.51 | 0.09 | 0.17 | -0.04 | -0.14 | 0.12 | 0.11 |
| PMS 1438 | *N* | 21 | 18 | 24 | 15 | 22 | 22 | 20 | 20 | 9 | 13 | 24 | 18 | 23 | 19 | 17 | 23 |
|  | A | 2 | 3 | 2 | 3 | 2 | 2 | 2 | 2 | 3 | 2 | 2 | 2 | 2 | 2 | 2 | 2 |
|  | *R*_S_ | 2.00 | 2.40 | 2.00 | 2.72 | 2.00 | 2.00 | 2.00 | 2.00 | 2.78 | 2.00 | 2.00 | 2.00 | 2.00 | 2.00 | 2.00 | 2.00 |
|  | *Pa* | 0.00 | 0.07 | 0.00 | 0.20 | 0.00 | 0.00 | 0.00 | 0.00 | 0.18 | 0.00 | 0.00 | 0.00 | 0.00 | 0.00 | 0.00 | 0.00 |
|  | *H*e | 0.51 | 0.50 | 0.51 | 0.56 | 0.51 | 0.51 | 0.51 | 0.51 | 0.58 | 0.52 | 0.51 | 0.51 | 0.51 | 0.49 | 0.52 | 0.51 |
|  | *H*o | 1.00 | 0.90 | 0.92 | 0.87 | 0.96 | 1.00 | 1.00 | 1.00 | 1.00 | 0.92 | 1.00 | 0.94 | 0.91 | 0.79 | 1.00 | 1.00 |
|  | *F*_IS_ | **-1.0** | -0.70 | **-0.84** | -0.58 | **-0.91** | **-1.00** | **-1.00** | **-1.00** | -0.80 | -0.85 | **-1.00** | **-0.89** | **-0.83** | -0.64 | **-1.00** | **-1.00** |
| PMS 1665 | *N* | 20 | 20 | 24 | 18 | 22 | 21 | 19 | 19 | 9 | 13 | 24 | 18 | 21 | 19 | 16 | 23 |
|  | A | 3 | 1 | 1 | 1 | 3 | 4 | 3 | 3 | 1 | 1 | 1 | 1 | 4 | 2 | 2 | 1 |
|  | *R*_S_ | 2.94 | 1.00 | 1.00 | 1.00 | 1.64 | 3.19 | 2.61 | 2.60 | 1.00 | 1.00 | 1.00 | 1.00 | 2.23 | 1.37 | 1.44 | 1.00 |
|  | *Pa* | 0.09 | 0.00 | 0.00 | 0.00 | 0.01 | 0.18 | 0.02 | 0.02 | 0.00 | 0.00 | 0.00 | 0.00 | 0.15 | 0.01 | 0.16 | 0.00 |
|  | *H*e | 0.59 | 0.00 | 0.00 | 0.00 | 0.09 | 0.52 | 0.49 | 0.45 | 0.00 | 0.00 | 0.00 | 0.00 | 0.18 | 0.05 | 0.06 | 0.00 |
|  | *H*o | 0.50 | 0.00 | 0.00 | 0.00 | 0.09 | 0.48 | 0.63 | 0.37 | 0.00 | 0.00 | 0.00 | 0.00 | 0.14 | 0.05 | 0.06 | 0.00 |
|  | *F*_IS_ | 0.15 | NA | NA | NA | -0.01 | 0.08 | -0.29 | 0.182 | NA | NA | NA | NA | 0.221 | 0 | 0 | NA |
| PMS 1694 | *N* | 21 | 20 | 24 | 18 | 22 | 19 | 19 | 20 | 9 | 13 | 24 | 18 | 23 | 18 | 18 | 23 |
|  | A | 3 | 3 | 2 | 3 | 3 | 4 | 3 | 3 | 4 | 4 | 3 | 3 | 4 | 4 | 3 | 2 |
|  | *R*_S_ | 2.84 | 2.58 | 1.95 | 2.71 | 2.23 | 2.69 | 2.46 | 2.35 | 3.52 | 3.34 | 2.29 | 2.32 | 2.83 | 2.74 | 2.35 | 1.30 |
|  | *Pa* | 0.00 | 0.01 | 0.00 | 0.00 | 0.00 | 0.00 | 0.00 | 0.00 | 0.00 | 0.00 | 0.00 | 0.00 | 0.00 | 0.00 | 0.00 | 0.00 |
|  | *H*e | 0.46 | 0.50 | 0.28 | 0.38 | 0.21 | 0.36 | 0.28 | 0.51 | 0.40 | 0.61 | 0.53 | 0.29 | 0.54 | 0.38 | 0.33 | 0.04 |
|  | *H*o | 0.43 | 0.10 | 0.33 | 0.33 | 0.23 | 0.42 | 0.32 | 0.80 | 0.33 | 0.31 | 0.50 | 0.22 | 0.52 | 0.11 | 0.17 | 0.04 |
|  | *F*_IS_ | 0.07 | **0.89** | -0.18 | 0.12 | -0.07 | -0.18 | -0.11 | -0.59 | 0.17 | 0.50 | 0.06 | 0.25 | 0.04 | 0.71 | 0.51 | 0.00 |
| PMS 2190 | *N* | 15 | 19 | 18 | 17 | 21 | 20 | 16 | 15 | 7 | 10 | 22 | 18 | 20 | 16 | 16 | 23 |
|  | A | 2 | 2 | 3 | 3 | 2 | 3 | 3 | 2 | 1 | 1 | 2 | 2 | 3 | 2 | 2 | 2 |
|  | *R*_S_ | 1.72 | 1.37 | 2.17 | 2.65 | 1.72 | 2.19 | 2.43 | 2.00 | 1.00 | 1.00 | 2.00 | 1.63 | 2.48 | 1.69 | 1.69 | 1.30 |
|  | *Pa* | 0.01 | 0.00 | 0.00 | 0.08 | 0.01 | 0.00 | 0.01 | 0.00 | 0.00 | 0.00 | 0.00 | 0.00 | 0.01 | 0.01 | 0.00 | 0.13 |
|  | *H*e | 0.13 | 0.10 | 0.21 | 0.43 | 0.14 | 0.23 | 0.43 | 0.43 | 0.00 | 0.00 | 0.50 | 0.11 | 0.31 | 0.12 | 0.12 | 0.04 |
|  | *H*o | 0.13 | 0.10 | 0.22 | 0.53 | 0.14 | 0.25 | 0.56 | 0.60 | 0.00 | 0.00 | 0.82 | 0.11 | 0.35 | 0.00 | 0.13 | 0.04 |
|  | *F*_IS_ | -0.04 | 0.00 | -0.07 | -0.25 | -0.05 | -0.09 | -0.31 | -0.40 | NA | NA | -0.68 | -0.03 | -0.14 | 1.00 | -0.03 | 0.00 |


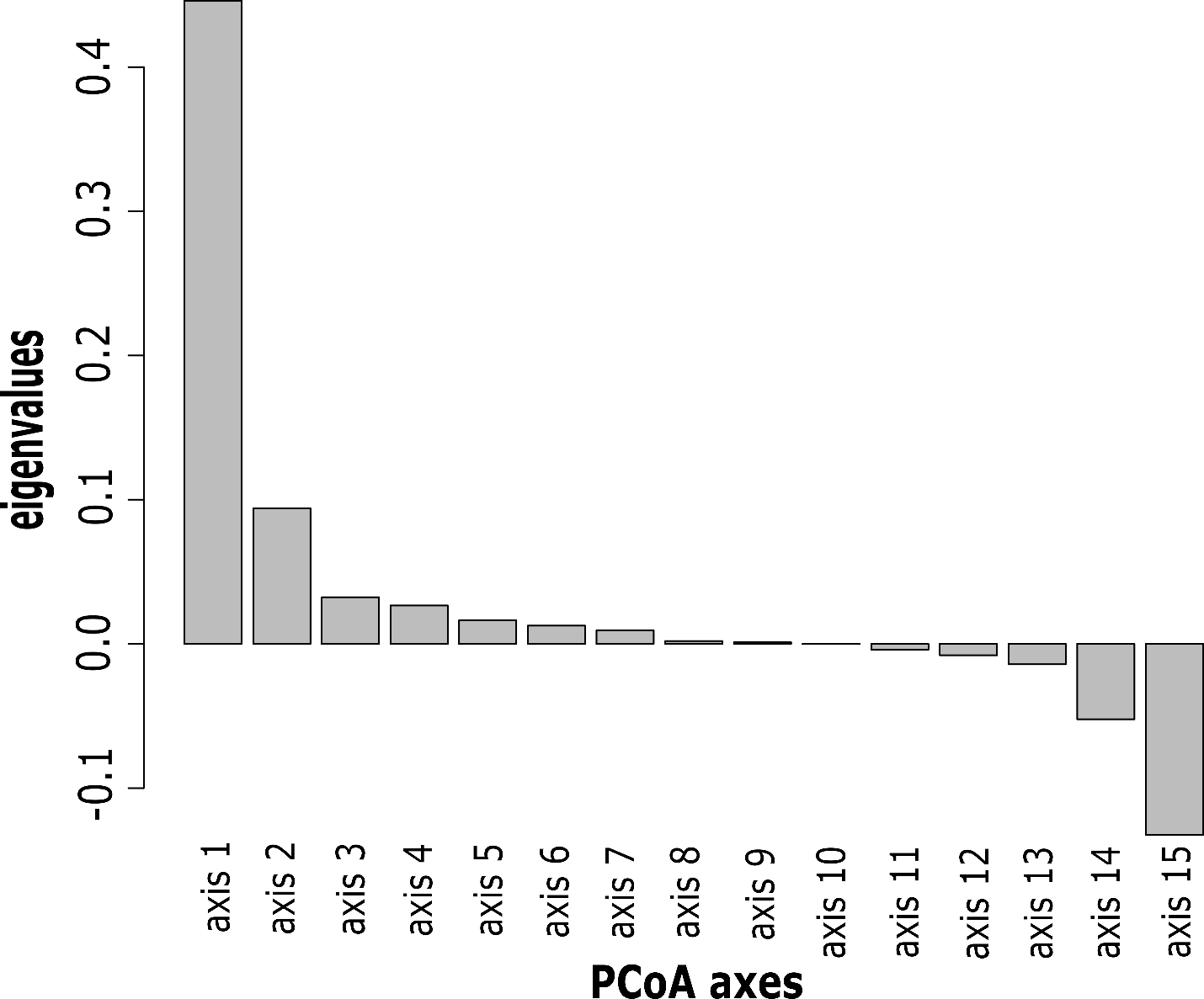


**Appendix S7**. A plot of the eigenvalues of the 15 axes in the Principal Coordinates Analysis (PCoA) of the pairwise *F*_ST_ genetic distance for the 16 sampled *Ivesia webberi* populations
